# Mannosylated STING Agonist Drugamers for Dendritic Cell-Mediated Cancer Immunotherapy

**DOI:** 10.1021/acscentsci.3c01310

**Published:** 2024-02-23

**Authors:** Dinh Chuong Nguyen, Kefan Song, Simbarashe Jokonya, Omeed Yazdani, Drew L. Sellers, Yonghui Wang, ABM Zakaria, Suzie H. Pun, Patrick S. Stayton

**Affiliations:** † Molecular Engineering & Sciences Institute, University of Washington, Seattle, Washington 98195, United States; ‡ Department of Bioengineering, University of Washington, Seattle, Washington 98195, United States

## Abstract

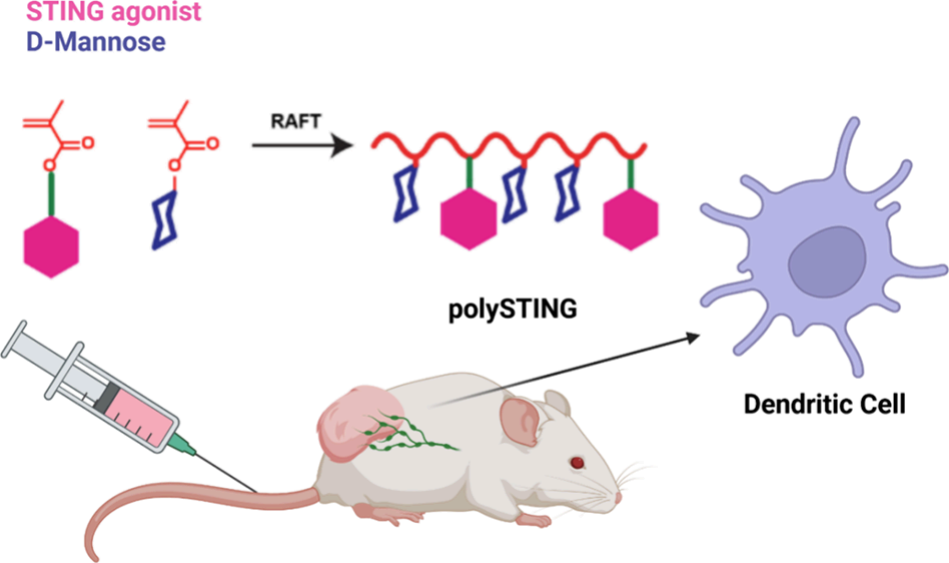

The Stimulator of Interferon Genes (STING) pathway is a promising target for cancer immunotherapy. Despite recent advances, therapies targeting the STING pathway are often limited by routes of administration, suboptimal STING activation, or off-target toxicity. Here, we report a dendritic cell (DC)-targeted polymeric prodrug platform (polySTING) that is designed to optimize intracellular delivery of a diamidobenzimidazole (diABZI) small-molecule STING agonist while minimizing off-target toxicity after parenteral administration. PolySTING incorporates mannose targeting ligands as a comonomer, which facilitates its uptake in CD206^+^/mannose receptor^+^ professional antigen-presenting cells (APCs) in the tumor microenvironment (TME). The STING agonist is conjugated through a cathepsin B-cleavable valine-alanine (VA) linker for selective intracellular drug release after receptor-mediated endocytosis. When administered intravenously in tumor-bearing mice, polySTING selectively targeted CD206^+^/mannose receptor^+^ APCs in the TME, resulting in increased cross-presenting CD8^+^ DCs, infiltrating CD8^+^ T cells in the TME as well as maturation across multiple DC subtypes in the tumor-draining lymph node (TDLN). Systemic administration of polySTING slowed tumor growth in a B16-F10 murine melanoma model as well as a 4T1 murine breast cancer model with an acceptable safety profile. Thus, we demonstrate that polySTING delivers STING agonists to professional APCs after systemic administration, generating efficacious DC-driven antitumor immunity with minimal side effects. This new polymeric prodrug platform may offer new opportunities for combining efficient targeted STING agonist delivery with other selective tumor therapeutic strategies.

## Abstract 2

We developed a novel polymer for targeted STING agonist drug delivery to dendritic cells, a key mediator of immunity, for cancer immunotherapy.

## 1 Introduction

Immune therapies that enlist patients’ endogenous immune systems are an effective and expanding strategy in treating cancer.^1^ While many of the recent innovations such as checkpoint inhibitors^2^ and CAR T cell therapies^3^ have focused on directly stimulating the adaptive immune system, therapeutics that engage the innate immune system provide an alternative approach to activating both adaptive and innate arms of immunity against cancer.^4^ The Stimulator of Interferon Genes (STING) pathway has emerged as a leading target. STING activation elicits a type-I interferon-driven response which induces a potent multifaceted innate and adaptive immune response.^5^ Systemic administration of STING agonists can initiate the cancer-immunity cycle.^6^ In a tumor-targeted context, initial STING activation in the tumor microenvironment (TME) provides an initial wave of immunogenic ablation through direct cytotoxicity or cell- or cytokine-mediated cytotoxicity. Resultant tumor antigens drain to secondary lymphoid organs, where they are presented by professional antigen-presenting cells (APCs) such as dendritic cells (DCs) to activate adaptive immunity with the help of STING-derived immunogenic cues (e.g., cytokines and costimulatory molecules). Activated adaptive immune cells mobilize toward tumors where they further induce immunogenic tumor cell killing to perpetuate the cycle.^7^ Indeed, successful STING activation can result in potent tumor suppression, eradication, or even long-term immunity to rechallenge/relapse.^8^

The potential in activating STING in cancer immunotherapy is clear, but drug delivery challenges have limited clinical translation of STING agonists. Canonical STING agonists such as 2′3′-cyclic GMP-AMP (2′3′-cGAMP) face significant systemic, cellular, and intracellular barriers in reaching the STING protein,^9^ necessitating either toxic high doses or the impractical intratumoral administration route.^10^ In addition to the development of alternative small-molecule STING agonists,^11−13^ multiple drug delivery platforms have been developed to address STING delivery challenges.^14^ Lipid micelles,^15^ polymersomes,^16,17^ liposomes,^18,19^ inorganic nanoparticles,^20^ or drug-conjugated polymeric particles^21^ have all been employed as systemic STING agonist carriers with potent responses.

Tumor-targeted STING delivery vehicles hold great promise. A reported tumor-targeted STING agonist antibody-drug conjugate (ADC) platform demonstrated an impressive safety profile and good efficacy.^22^ However, this platform encounters inherent issues with target availability and scale-up manufacturability.^23^ In addition, low-level STING activation in tumors can induce immunosuppressive factors such as indoleamine-2,3-deoxygenase (IDO), causing pathogenesis.^24^ As tumor cells can downregulate STING,^25^ agonist delivery to tumor cells can be either ineffective or counterproductive through this mechanism. Conversely, overactivation of STING can result in unproductive nonimmunogenic cell ablation,^26,27^ rendering patients vulnerable to relapse or metastasis. In light of this, targeted STING delivery to intratumoral APCs such as dendritic cells (DCs) may induce antitumor immunity without the aforementioned shortcomings. In tumor rejection models with or without immunotherapy, DC subsets such as type-1 conventional DCs (cDC1) play indispensable roles in antigen transport, cross-priming, and lymphocyte recruitment and maintenance in the TME.^28−32^ STING activation in DCs strongly induces DC maturation,^33^ and activation in cDC1 was crucial to the function of an intratumoral viral vector STING delivery platform.^27^ We hypothesize that a STING delivery platform targeting tumoral APCs can induce potent antitumor responses with minimal toxicity. A Clec9a^+^ DC-targeted STING delivery platform showcased this possibility.^34^

Here, we report a DC-targeted STING polymeric prodrug platform that elicits a robust DC-driven antitumor response after systemic intravenous administration. We synthesized a polymerizable STING agonist prodrug monomer by conjugating a diamidobenzimidazole (diABZI)-type agonist,^11^ “STING Agonist-3”, to a polymerizable methacrylate via an intracellular cathepsin-cleavable valine-alanine (VA) linker. The monomer was copolymerized with a targeting mannose methacrylate monomer^35^ to produce the targeted macromolecular STING agonist prodrug “drugamer”, termed polySTING. We showed that polySTING targets CD206^+^/mannose receptor^+^ professional APCs in the TME, activates STING *in vivo*, and is well-tolerated. We confirmed polySTING’s efficacy in an aggressive “cold” B16-F10 murine melanoma model. We examined polySTING’s mechanism in the same model and found a strong DC-driven antitumor immune response. Specifically, polySTING strongly enhanced CD8^+^ cDC1 responses along with TME T-lymphocyte infiltration and CD8^+^ T cell activation while promoting DC maturation in the tumor-draining lymph nodes (TDLN). PolySTING also reduced tumor growth kinetics in the aggressive 4T1 breast cancer model of a different murine strain, demonstrating the utility of polySTING as a standalone systemic cancer immunotherapy or especially as a candidate for combination immune-therapy strategies.

## 2 Results and Discussion

### Synthesis and Characterization of PolySTING

**Figure 1:**
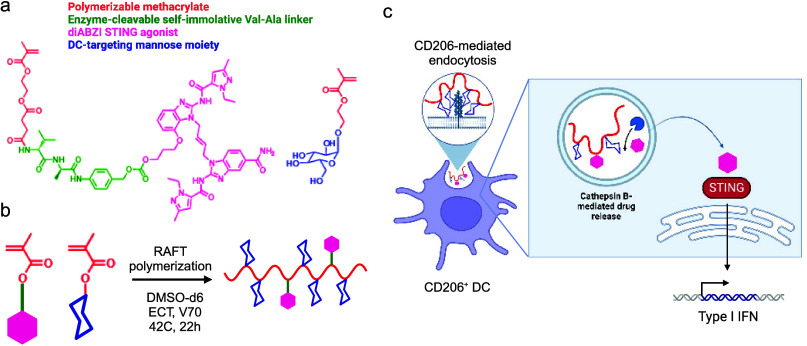
Design of a polySTING polymeric prodrug. (a) Structure of the enzyme-cleavable methacrylate-based STING agonist prodrug monomer and mannose ethyl methacrylate monomer. (b) Schematic of polySTING synthesis by RAFT polymerization. (c) Schematic of polySTING uptake by CD206^+^ DCs, endosomal prodrug cleavage and agonist release, and STING activation in DCs. Created with BioRender.com.

“STING Agonist-3” was selected for our studies because it shows excellent activity^11^, and has a single hydroxyl group amenable to the synthesis of the polymerizable prodrug monomer. The synthetic scheme for the STING Agonist-3 prodrug monomer is summarized in Figure 1a (more detail in Scheme S1). Using the polymerizable mono-2-(methacryloyloxy)ethyl succinate (SMA) moiety, the prodrug monomer was synthesized by incorporating the cathepsin B-cleavable Val-Ala (VA) linker with a self-immolative para-aminobenzyl alcohol (PABA) moiety that has been validated in the human antibody-conjugate field^36^ to yield SMA-Val-Ala-PABA-STING Agonist-3 (SVA-PAB-STING). The prodrug monomer product was validated using ^1^H NMR and mass spectrometry (Figures S1 and S2). We then synthesized the targeted polymer prodrug “drugamer” polySTING by copolymerizing SVA-PAB-STING with mannose ethyl methacrylate (ManEMA) (Figure 1b, characterization in Figures S3–S5). PolySTING has an average Mw of 12 kDa, with average degrees of polymerization (DP) of 35 for ManEMA and 2 for SVA-PAB-STING. We confirmed by dynamic light scattering that polySTING is highly soluble in aqueous solutions as unimers of up to 200 mg/mL in PBS (Figure S6). PolySTING is thus designed for internalization by receptor-mediated endocytosis in CD206^+^ APCs, followed by endosomal cathepsin cleavage and release of the membrane-permeable diABZI-type STING Agonist-3 that can activate the cytosolic STING protein in these APCs (Figure 1c).

### PolySTING Targets APCs in the TME and Activates STING

**Figure 2:**
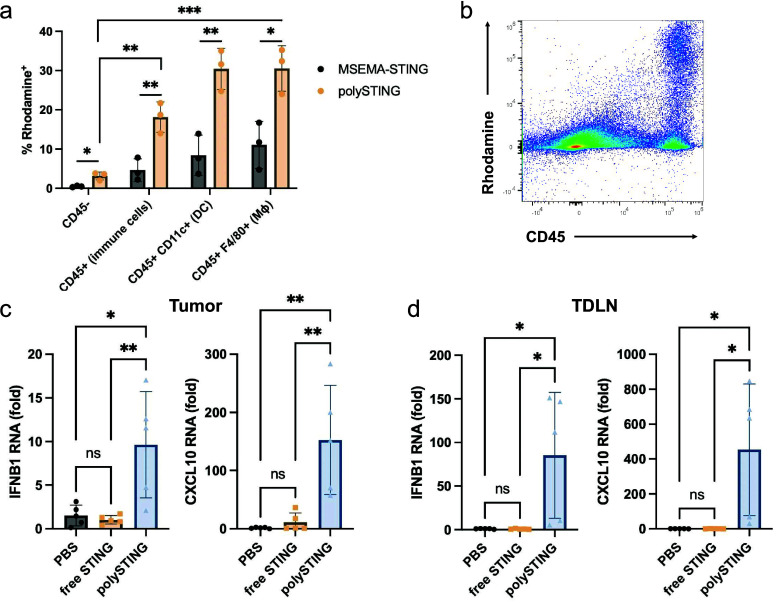
PolySTING targets immune cells and activates the STING pathway. (a) B16-F10 tumor-bearing C57BL/6 mice were treated with polySTING-Rh or MSEMA-STING-Rh intravenously, and tumors (100–200 mm^3^) were harvested 30 min post-injection. Percentage of rhodamine-positive cells in different cell subsets was quantified by flow cytometry (*N* = 3). (b) Representative flow dot plot of rhodamine and CD45 expression in a tumor. (c, d) Expression levels of interferon-stimulated genes IFNB1 and CXCL10 in the tumor and TDLN 4 h post-treatment by RT-qPCR (*N* = 5). Gene expression was normalized to the GAPDH housekeeping gene. Data are represented as mean ± SD (a). An unpaired *t* test was used to compare polySTING and MSEMA-STING under different cell subsets. One-way ANOVA with a posthoc Tukey HSD test was used to compare the polymer uptake in different cell subsets after polySTING treatment (*p ≤ 0.05, **p ≤ 0.01, and ***p ≤ 0.001). (c, d) Statistical analysis was performed using a one-way ANOVA with posthoc Tukey HSD test (*p ≤ 0.05, **p ≤ 0.01).

In our preliminary pharmacokinetic characterization in B16-F10 tumor-bearing mice, we observed that the intravenous administration of polySTING resulted in higher plasma and tumor STING agonist concentrations compared to free STING agonist 30 min after administration (Table S1). These results motivated us to investigate the professional APC-targeting selectivity of polySTING in the tumor after intravenous administration, as these cells play pivotal roles in antitumor responses^37,38^ and may be targeted by mannose through the CD206 receptor. To this end, a fluorescent rhodamine-labeled polySTING (polySTING-Rh) was synthesized alongside a nontargeted control substituting ManEMA with the hydrophilic non-glycan 2-methylsulfinyl ethyl methacrylate (MSEMA-STING-Rh, NMR characterization in Figure S7). PolySTING-Rh and MSEMA-STING-Rh were administered to B16-F10-bearing C57BL/6 mice, and rhodamine-positive cell subsets in the tumor were characterized. PolySTING-Rh was shown to selectively target immune cells (CD45^+^) including DCs (CD11c^+^) and macrophages (F4/80^+^), which all have a significant fraction of polySTING-Rh-positive cells, unlike nonimmune CD45^–^ cells (Figure 2a,b). MSEMA-STING-Rh exhibited much less uptake in both immune cell subsets compared to polySTING (Figure 2a). We still observed the preferential uptake of MSEMA-STING in macrophages and DCs, but the difference compared to CD45 cells is less pronounced and explainable through inherent phagocytic activity. We observed similar results 4 h after treatment (Figure S8). PolySTING therefore has a unique TME distribution profile compared to prior nontargeted STING agonist formulations, which all have significant uptake in CD45^–^ cells.^15,16,21^ Thus, by transforming the STING agonist to a mannosylated macromolecular prodrug, we successfully restricted STING agonist delivery to immune cells in the TME.

STING activation was also assessed via the expression of STING-related genes IFNB1 and CXCL10 in the tumor and the tumor-draining lymph nodes (TDLNs). Tumor and TDLN STING gene expression was sampled 4 h after a single treatment. PolySTING elicited a significant increase in both IFNB1 and CXCL10 expression in the tumor compared to free STING agonist and untreated mice (Figure 2c). PolySTING generated a 9.3-fold increase in IFNB1 expression and a 13-fold increase in CXCL10 expression compared to free STING agonist. In the TDLN, polySTING induced a 111-fold increase in IFNB1 expression and a 383-fold increase in CXCL10 expression (Figure 2d).^39,40^ These results establish that polySTING preferentially targets APCs, resulting in STING pathway activation in the TME and in the TDLNs.

Because polySTING was shown to localize to both macrophages and DCs in the TME, downstream activation markers were investigated in each cell type. In macrophages, STING activation has been reported to drive polarization toward the pro-inflammatory M1 phenotype over the tolerogenic, tumorigenic M2 phenotype.^41,42^ Surprisingly, polySTING slightly increased M1 marker CD80 expression in tumor-associated macrophages (TAMs) compared to free STING agonist and untreated mice but had no effect on M2 marker CD206 expression (Figure S9). We investigated this trend *in vitro* with bone marrow-derived macrophages (BMDM) incubated with STING agonists and formulations. Similarly, STING treatments increased the expression of M1 marker CD86 in M2 macrophages but did not change the expression level of M2 markers CD206 and CD163 (Figure S10). RT-qPCR showed increased NOS2 (M1-related) expression and decreased ARG1 (M2-related) expression in polySTING-treated cells but only ARG1 downregulation for free agonist (Figure S11). These results suggest that polySTING could drive macrophage polarization toward the pro-inflammatory M1 type. In addition, polySTING induced significant DC maturation across all markers (CD86, CD80, and CD40) in the DC-enriched TDLN (Figure S12). The potent DC response suggests the potential for a strong DC-mediated adaptive antitumor immune response. We therefore moved to therapeutic evaluation *in vivo*.

### Systemic Therapy of PolySTING Is Tolerable and Results in the Potent Suppression of Melanoma Growth

We next investigated the organ-level biodistribution of polySTING using polySTING-Rh in the same B16-F10 model. At 30 min post-intravenous injection, we observed the highest polySTING-Rh signal in the liver, followed by the tumor and spleen (Figure S13). As aberrantly activated liver macrophages/Kupffer cells can cause liver damage, we next investigated liver toxicity. We intravenously administered polySTING or free STING agonist to C57BL/6 mice twice, 3 days apart, and sampled serum levels of alanine aminotransferase (ALT) and aspartate aminotransferase (AST) as markers of hepatotoxicity. Neither the intravenous administration of polySTING nor the free drug elevated ALT or AST levels above saline treatment (Figure S14). In addition, neither treatment resulted in significant weight loss, while polySTING exhibited dose-dependent IFNβ stimulation (Figures S15 and S16).

**Figure 3:**
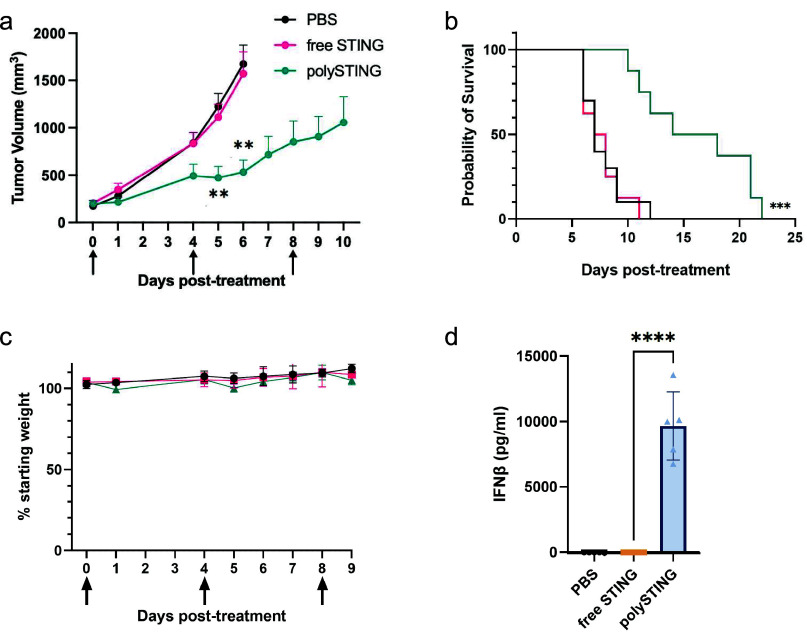
PolySTING inhibits tumor growth and prolongs survival in tumor-bearing mice. (a) Tumor growth curve in B16-F10 tumor-bearing C57BL/6 mice. B16-F10 cells were inoculated on day −8, and STING treatments were given on days 0, 4, and 8 through intravenous injections. Data are represented as mean ± SEM (*N* = 8–10). Statistical analysis was performed using mixed effects of the analysis of two-way ANOVA with Tukey’s multiple comparisons test (**p ≤ 0.01, between free STING and polySTING). (b) Kaplan–Meier survival curve (*N* = 8–10). Survival analysis was performed using the log-rank test (***p ≤ 0.001). (c) Weight change in B16-F10 tumor-bearing C57BL/6 mice. Data are represented as the mean ± SD (*N* = 8–10). (d) Plasma IFNβ level in 4T1 tumor-bearing BALB/c mice 4 h post-treatment with free STING and polySTING (*N* = 5). Data are represented as the mean ± SD. Statistical analysis was performed using one-way ANOVA with a posthoc Tukey HSD test (****p ≤ 0.0001).

These indications of tolerability permitted the subsequent evaluation of polySTING’s anticancer efficacy in the aggressive, poorly immunogenic murine melanoma model B16-F10.^43^ PolySTING significantly inhibited B16-F10 tumor growth and prolonged survival compared to vehicle control (Figure 3a,b, individual curves in Figure S17). Free-form STING Agonist-3 did not yield any therapeutic effect over control. The diABZI family from which STING Agonist-3 was derived already has an efficacy track record in the CT-26 colon cancer model;^11^ these striking results reflect the aggressiveness of the B16-F10 model and highlight the therapeutic utility of polySTING. In the same study, a stronger IFNβ response and higher weight loss upon either polySTING or free drug treatment was observed compared to healthy mice, likely due to inflammation in the tumor causing weight loss from ablation (Figure 3c,d compared with Figures S15 and S16). Weight loss increased with subsequent administration; however, it remained below 10% loss up to the third injection, a treatment regime comparable to many other reported systemic STING delivery platforms.^16,18,21^

### Examination of PolySTING-Treated B16-F10-Bearing Mice Reveals a T-Cell-Inflamed TME Maintained by CD8^+^ DCs

**Figure 4:**
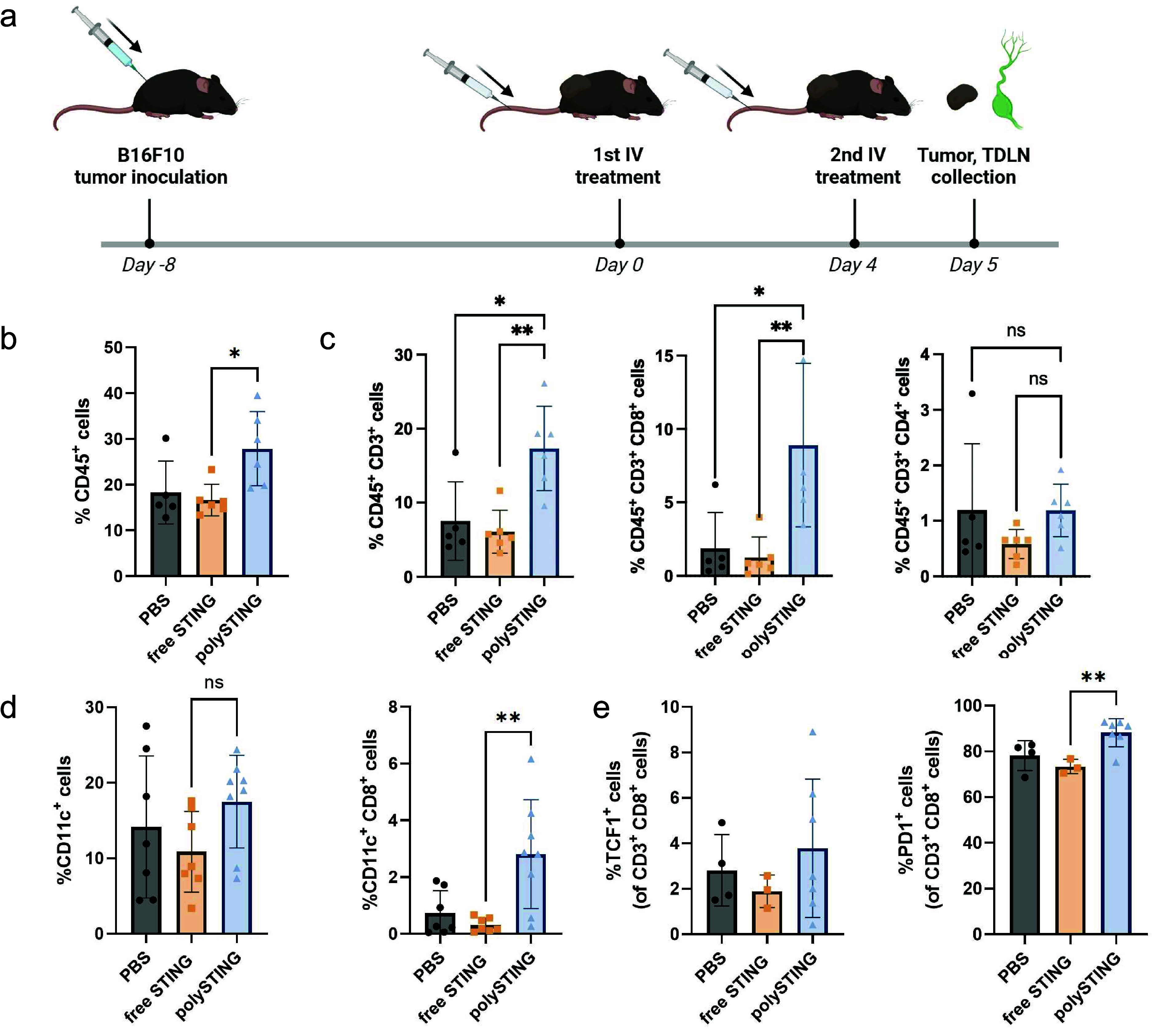
PolySTING induces immune cell infiltration into the tumor. (a) Schematic of polySTING treatment schedule. C57BL/6 mice were inoculated with B16-F10 cells on day −8. Treatment was given on day 0 and day 4 intravenously. Tumor and TDLN were harvested 24 h after the second injection. (b) Percentage of CD45^+^ immune cells in the tumor by flow cytometry (*N* = 5–6). (c) Percentage of total T cells, CD8^+^ T cells, and CD4^+^ T cells in the tumor by flow cytometry (*N* = 5–6). (d) Percentage of total DCs and CD8^+^ DCs in the tumor by flow cytometry (*N* = 7–8). (e) Percentage of TCF1^+^ CD8^+^ T cells and PD1^+^ CD8^+^ T cells in the tumor by flow cytometry. Data are represented as the mean ± SD. Statistical analysis was performed using a one-way ANOVA with posthoc Tukey HSD test (*p ≤ 0.05, **p ≤ 0.01, ns: not significant). Panel a was created with BioRender.com.

To better understand the mechanism behind polySTING’s efficacy, the immune cell population in the B16-F10 TME was examined after the second injection (Figure 4a). PolySTING induced more CD45^+^ immune cell infiltration into the tumor compared to the free drug (Figure 4b). As T cells are key contributors in antitumor immunity,^44^ we first looked at the T-cell infiltration (gating scheme in Figure S18). In line with the tumor suppression results, polySTING treatment, but not free STING agonist, induced significantly more T-cell infiltration (2.3-fold) in the TME than in untreated mice (Figure 4c). More importantly, polySTING treatment increased the percentage of CD8^+^ T cells in the TME by 4.8-fold and could partially explain the antitumor effect of polySTING.

As tumor-infiltrating T cells could still be subject to immunosuppression and therapy failure,^45^ the DC subset composition was next examined (gating scheme in Figure S19). cDC1s are critical mediators of antigen transport to TDLNs^46^ and chemoattractant producers in the TME (i.e., CD103^+^ DCs)^30^ and exhibit antigen cross-presentation to T cells for both priming and maintenance (i.e., CD8^+^ DCs).^29^ PolySTING increased CD8^+^ DCs in the TME compared to untreated or free drug groups but not the overall DC population relative to all cells (Figure 4d). To our knowledge, this CD8^+^ DC response without the use of checkpoint blockade therapy has not been reported elsewhere and may represent a novel mechanism for targeted STING immunotherapy. Tumoral DCs have been recognized as crucial to maintaining T-cell activity in the TME through antigen presentation and co-stimulation.^32^ Given CD8^+^ DCs’ capability for cross-presentation to cytotoxic lymphocytes,^47^ their population increase in polySTING-treated groups compared to free drug could better maintain tumoral CD8^+^ T-cell activity. The increased CD8^+^ DC population in the TME without a change in the overall CD11c^+^ DC population may represent either the polarization of CD11c^+^ cells toward CD8^+^ phenotypes or a migratory equilibrium between CD8^+^ DCs and other subsets. This is consistent with reports that STING activation and/or its resultant IFN response stimulates CD8^+^ DC responses.^27,48,49^ There were no differences in CD103^+^ DC populations between treatment groups (Figure S20).

The quality of infiltrating T-cells was also examined through an analysis of PD-1 and TCF-1 expression (gating scheme in Figure S21). PD-1 is a marker of tumor-reactive CD8^+^ T cells in melanoma.^50^ TCF-1 is a marker indicative of stem-like constitutively activated T-cells (i.e., exhausted T-cells in the antigen-rich TME) capable of functional rescue^51^ and is mechanistically associated with positive response checkpoint blockade therapy.^52^ PolySTING increased the fraction of “tumor-reactive” PD-1^+^ CD8^+^ T cells while the free-form STING agonist did not (Figure 4e). The increase in PD-1^+^ CD8^+^ T cells may be a consequence of increased T-cell infiltration into the tumor and the heightened persistent antigen exposure associated with it.^53^ There was no difference in TCF-1 expression among all treatment groups, though the response trended upward for polySTING (Figure 4e).

### PolySTING Also Positively Impacts DC Function in the TDLN

**Figure 5:**
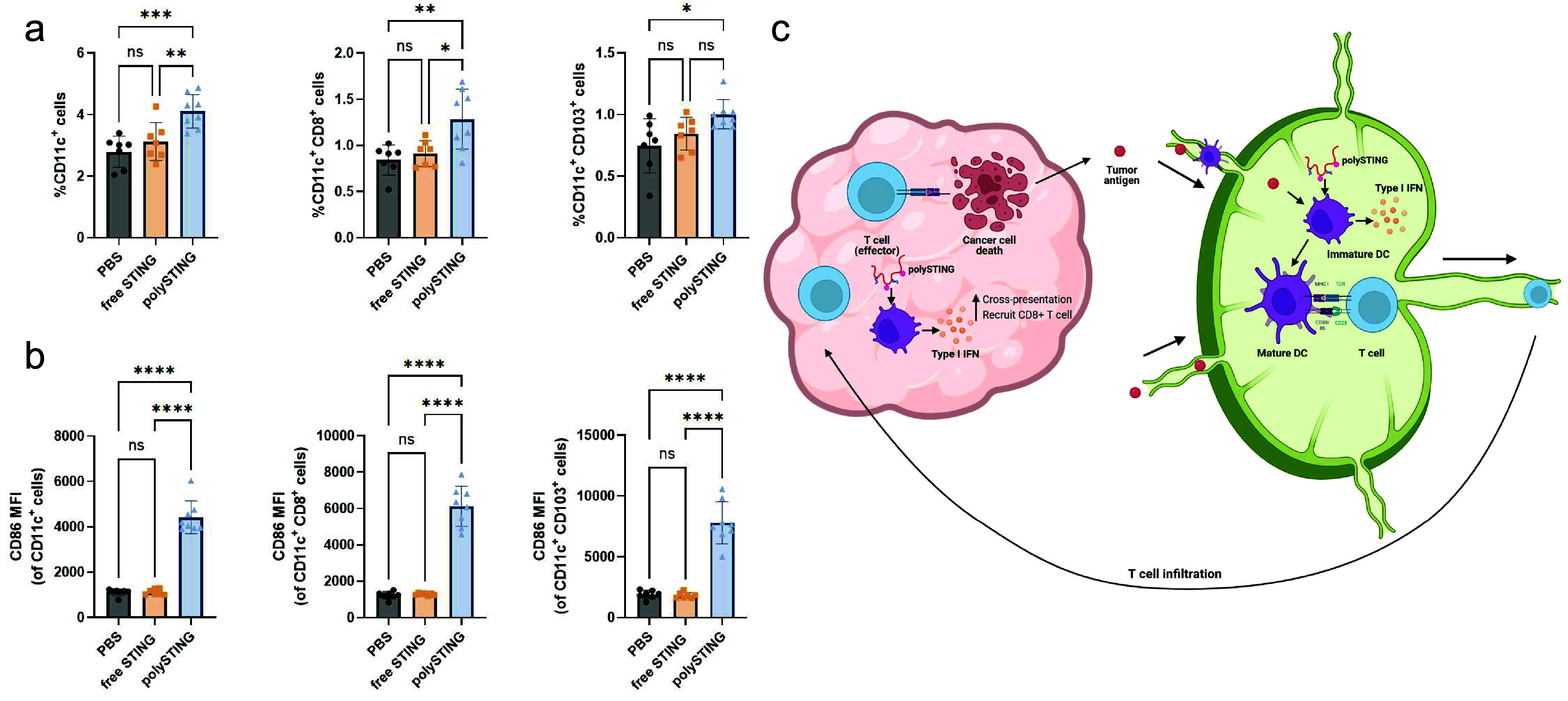
PolySTING induces DC proliferation and maturation in the TDLN. (a) Percentage of total DCs, CD8^+^ DCs, and CD103^+^ DCs in the TDLN by flow cytometry (*N* = 7–8). (b) CD86 expression on total DCs, CD8^+^ DCs, and CD103^+^ DCs in the TDLN by flow cytometry (*N* = 7–8). (c) Schematic of the polySTING mechanisms in the tumor and TDLN. Data are represented as the mean ± SD. Statistical analysis was performed using one-way ANOVA with a posthoc Tukey HSD test (*p ≤ 0.05, **p ≤ 0.01, ***p ≤ 0.001, and ****p ≤ 0.0001; ns, not significant). Panel c was generated with BioRender.com.

Having demonstrated potent downstream effects of polySTING, we next looked upstream at the TDLNs where T-cell priming takes place (gating scheme in Figure S22). In contrast to the TME, there is a noticeable increase in CD11c^+^ DCs in polySTING-treated TDLNs as well as cDC1 subsets CD8^+^ and CD103^+^ DCs (Figure 5a). In addition, all DC subsets had a substantial increase in the CD86 maturation marker (Figure 5b). Consistent with previous data, the free-form STING agonist did not induce any appreciable change. The increased maturation marker expression could be due to a combination of type I IFN production due to STING activation and exposure to tumor antigen.^54,55^ The indispensable role of secondary lymphoid organ (SLO)-resident CD8^+^ DCs in priming cytotoxic T-lymphocytes (CTLs) and subsequent cellular responses to both tumors and pathogens is well-established;^29^ thus, their expansion brought about by polySTING indicates stronger CD8^+^ T cell priming. The increase in CD103^+^ DCs in the TDLN of polySTING-treated animals compared to control animals is in line with a report showing CD103^+^ DCs passing tumor antigen to CTL-priming CD8^+^ DCs via synaptic transfer after migration to TDLNs.^56^ CD103^+^ DCs are also potent activators of naive CD8^+^ T-cells through cross-presentation, complementing this effect.^57^ Thus, a more complete image of polySTING’s proposed mechanism of action emerges (Figure 5c): DC-targeted polySTING activates the STING pathway in DCs and results in type I IFN production.^6^ Type I IFNs activate DCs and induce the survival and proliferation of CD8^+^ DCs.^54^ Tumor antigens travel to the TDLNs either by themselves or transported by migratory DCs.^58^ Migratory CD103^+^ DCs take up and transport tumor antigen to the TDLNs for cross-presentation through either self- or antigen transfer to CD8^+^ DCs, both resulting in effective T-cell priming. Activated T cells travel to the tumor through the vasculature, and type-I IFNs promote T-cell infiltration in the TME.^59^ The strong cytotoxic CD8^+^ T-cell response results in a strong antitumor effect. More antigens are generated and transported to the TDLNs to further activate DCs and T cells, perpetuating the cancer-immunity cycle.^7^

### Validation of Systemic PolySTING Therapeutic Efficacy in the 4T1 Breast Cancer Model

**Figure 6:**
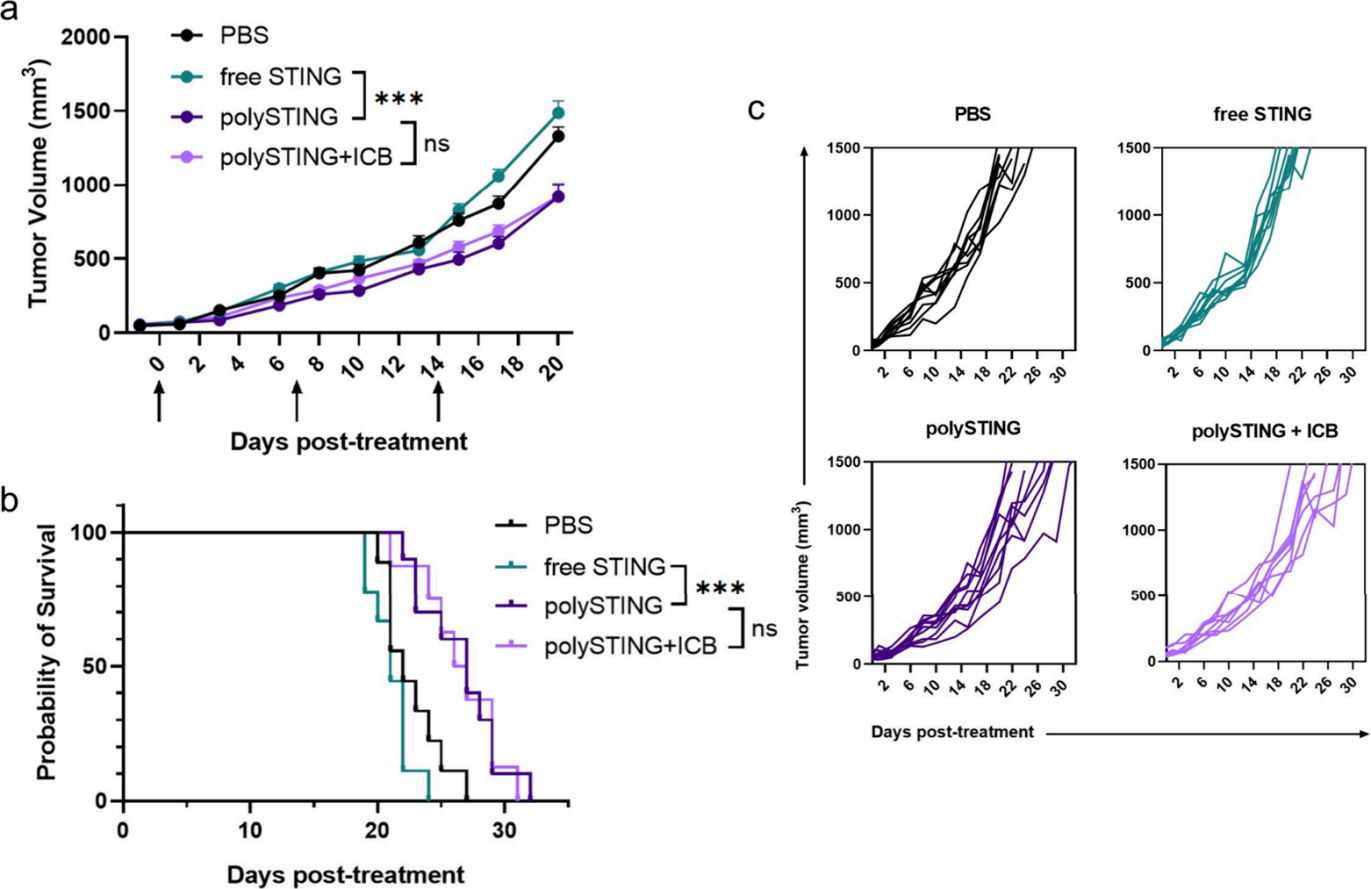
PolySTING shows antitumor efficacy in the 4T1 model. (a) Tumor growth curve in 4T1 tumor-bearing BALB/c mice. 4T1 cells were inoculated on day −8, and STING treatments were given on days 0, 7, and 14. ICB was given on days 1, 8, and 15. Data are represented as the mean ± SEM (*N* = 8–10). Statistical analysis was performed using mixed-effects analysis of two-way ANOVA with Tukey’s multiple comparisons test (***p ≤ 0.001, ns: no significance, on day 20). (b) Kaplan–Meier survival curve (*N* = 8–10). Survival analysis was performed using the log-rank test (*** *p* ≤ 0.001; ns, no significance). (c) Individual growth curves.

To further demonstrate the utility of polySTING, we evaluated the efficacy in another solid tumor type in a different mouse strain. We chose the 4T1 orthotopic breast cancer model in BALB/c mice also for its low immunogenicity and late-stage aggressiveness.^43^ In addition, 4T1’s resistance to checkpoint blockade therapy in our experience and polySTING’s strong induction of PD-1^+^ CD8^+^ T cells make it a good candidate to test for synergy with anti-PD-1. We also observed tumor growth inhibition and survival benefits with systemic polySTING treatment in this model, with similar weight loss (Figure S23) albeit with a more modest therapeutic effect (Figure 6). Consistent with our previous data, the free-form STING agonist did not exert any measurable therapeutic effect. Interestingly, co-therapy with anti-PD-1 did not further inhibit tumor growth or improve survival. Activated DCs are known to express PD-L1, which affects the response to checkpoint blockade therapy.^60,61^ As tumor-activated DCs have been shown to accumulate in solid tumors,^62^ it is possible that polySTING’s DC-centric therapeutic modality generated a tumoral reservoir of DC-associated PD-L1 that outlasts anti-PD-1 antibodies^63^ and PD-1-blocked T cells.^32,64^ Conversely, as 4T1 tumors have been reported to express only low levels of PD-L1,^65^ it is possible that the tumor model inherently does not benefit from PD-1/PD-L1 immunotherapy. This is consistent with other reports of systemic STING delivery therapy in the 4T1 model showing little to no benefit with anti-PD-1 therapy.^21^ Further mechanistic studies in the 4T1 model as well as therapeutic studies in different tumor models will be necessary to delineate this phenomenon. Regardless, polySTING’s efficacy in two different aggressive, nonimmunogenic tumor models across two different mouse strains demonstrates its strong utility as a systemic immunotherapy.

## 4 Conclusions

We have developed a novel polymeric platform, polySTING, to deliver STING agonists to DCs through systemic administration. PolySTING was primarily taken up by professional APCs, including DCs, in the TME and resulted in systemic STING activity. PolySTING drove a strong DC-directed immune response through DC activation in the TDLN and the induction of cross-presenting CD8^+^ DCs in both the TDLN and TME. We observed significant therapeutic efficacy in two distinct aggressive nonimmunogenic tumor models with a good safety profile with polySTING as an intravenous immunotherapy. PolySTING is a novel platform for cancer immunotherapy and highlights the potential of targeted STING delivery to DCs.

## Supporting Information Available

Material and syntheses (STING monomer and polymer syntheses), characterization and experimental methods, and supporting figures and tables (Figures S1–S23, Table S1) (PDF)
