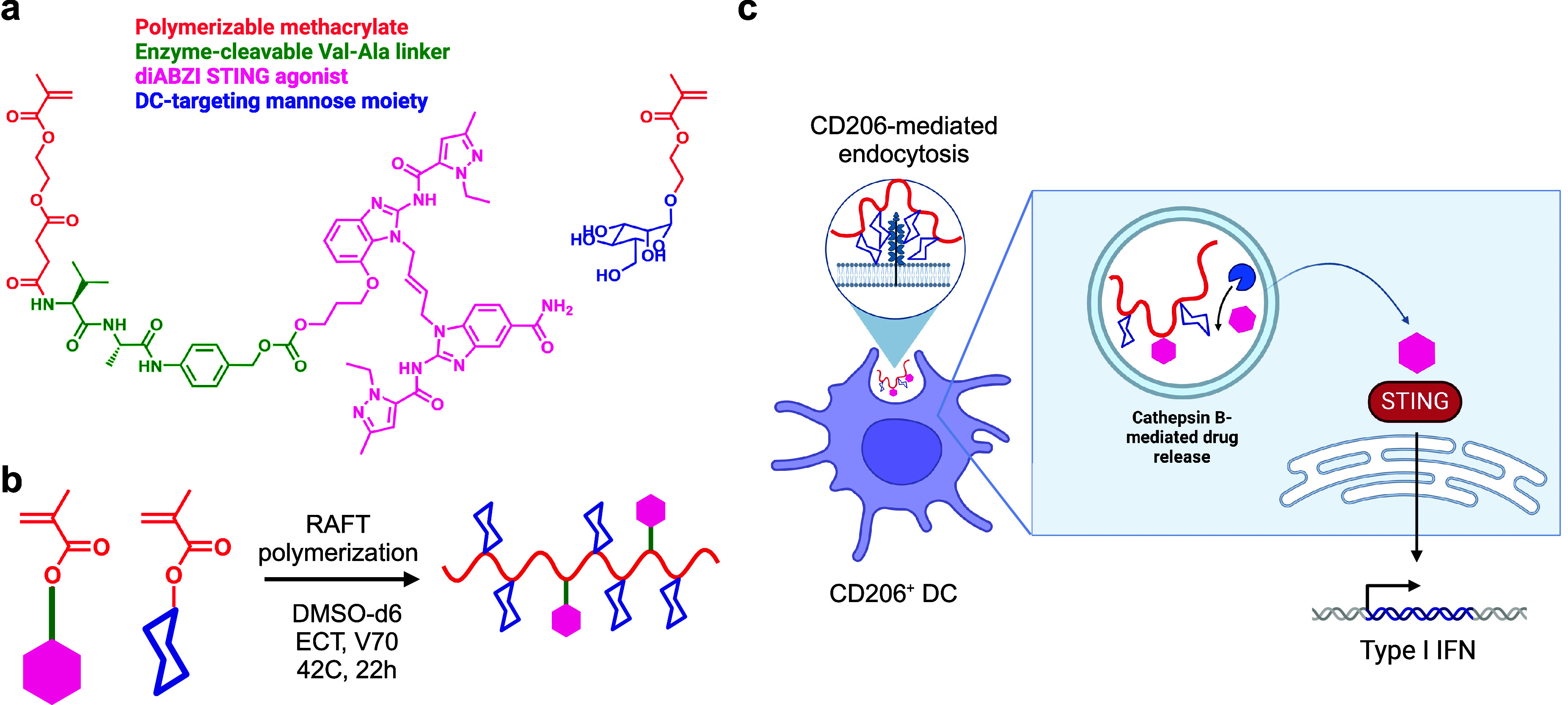# Correction to “Mannosylated STING Agonist Drugamers for Dendritic Cell-Mediated Cancer Immunotherapy”

**DOI:** 10.1021/acscentsci.4c00532

**Published:** 2024-04-16

**Authors:** Dinh Chuong Nguyen, Kefan Song, Simbarashe Jokonya, Omeed Yazdani, Drew L. Sellers, Yonghui Wang, ABM Zakaria, Suzie H. Pun, Patrick S. Stayton

The structure of D-mannose methacrylate in our publication was drawn incorrectly. Specifically, the hydroxyl at the C2 position was drawn in the equatorial position when it should be in the axial position as appropriate for mannose. This affected Figure 1 in the main text and Figure S3 in the Supporting Information. The corrected Figure 1 and Supporting Information file is included in this Correction. We apologize for any confusion that our mistake may have caused, and we would like to thank Prof. Sebastien Vidal and the Editorial staff of *ACS Central Science* for bringing this to our attention.

**Figure 1:**