# Supplementary material for: Correction to “Mannosylated STING Agonist Drugamers for Dendritic Cell-Mediated Cancer Immunotherapy”
Source: ACS Cent Sci. 2024 Apr 16;10(5):1117. doi: 10.1021/acscentsci.4c00532 (PMC11117684; doi:10.1021/acscentsci.4c00532)
Supplement: Supplementary file 1 — oc4c00532_si_001.pdf [file oc4c00532_si_001.pdf]

Supplementary Information - Mannosylated STING Agonist Drugamers For Dendritic Cell-Mediated Cancer Immunotherapy

Dinh Chuong Nguyen, Kefan Song, Simbarashe Jokonya, Omeed Yazdani, Drew L. Sellers, Yonghui Wang, ABM Zakaria, Suzie H. Pun, Patrick S. Stayton**

Department of Bioengineering and Molecular Engineering & Sciences Institute, University of Washington, USA

Email of co-corresponding authors (respectively): spun@uw.edu, stayton@uw.edu

Contents:

1) **Materials and Syntheses**
2) **Characterization and Experimental Methods**
3) **Supporting Figures and Tables**
4) **References**

1. Materials and Syntheses

Safety Statement

No unexpected or unusually high safety hazards were encountered in this work.

Materials

SMA N-hydroxysuccinimide ester (SMA-NHS, compound 2, **Scheme S1**) was synthesized as previously described[1]. 2-(methylsulfinyl)ethyl methacrylate (MSEMA) was synthesized as previously described[2]. The STING agonist "STING Agonist-3" (CAS 2138299-29-1) was purchased from MedChemExpress (Catalog #HY-103665). L-Valyl-L-Alanine (H-Val-Ala-OH) was purchased from Combi-Blocks. RAFT chain transfer agent 4-(((ethylthio)carbonothioyl)thio)-4-cyanopentanoic acid (ECT) and the monomer mannose ethyl methacrylate (ManEMA) were purchased from Omm Scientific. The rhodamine monomer, methacryloylethyl thiocarbamoyl rhodamine B (RhMA) was purchased from PolySciences Inc. The initiator 2,2'-azobis(4-methoxy-2,4-dimethylvaleronitrile) (also known as V70) was purchased from Wako Fujifilm. N-Ethoxycarbonyl-2-ethoxy-1,2-dihydroquinoline (EEDQ) and 4-nitrophenol chloroformate (PNP-Cl) was purchased from Chem-Impex International Inc.. 4-aminobenzyl alcohol (PABA) was purchased from Tokyo Chemical Industries. Diisopropylethylamine (DIPEA), triethylamine (TEA) and dimethylaminopyridine (DMAP) were purchased from Sigma-Aldrich. Dimethylformamide (DMF), tetrahydrofuran (THF), dichloromethane (DCM) and chloroform were purchased from Fisher. Argon gas was purchased from Linde Gas & Equipment Inc. All reagents mentioned above were used as received.

Anhydrous deuterated dimethyl sulfoxide (DMSO-d6, > 99%) was purchased from Sigma-Aldrich and stored with activated molecular sieves. Buffers were prepared in-house using endotoxin-free water and salts purchased from Fisher Scientific. All antibodies were purchased from BioLegend, with the exception of anti-TCF1-PE and anti-PD1-eFluor450.

Synthesis of STING agonist prodrug monomer

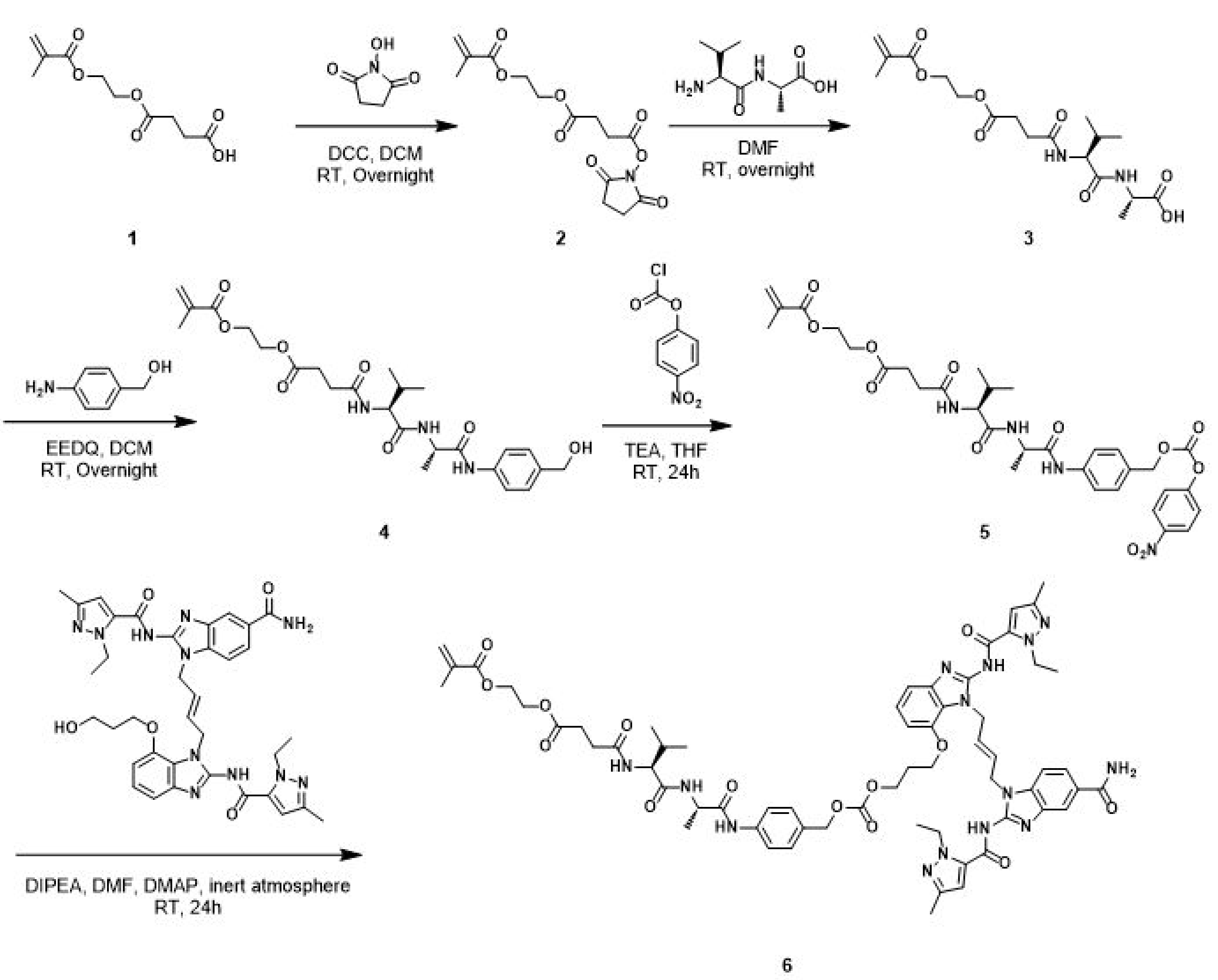

Scheme S1 - Synthesis of STING agonist prodrug monomer

Synthesis of SVA (Compound 3)

H-Val-Ala-OH (1.81 g, 9.63 mmol, 1 eq) was dissolved in DMF (22 mL). SMA-NHS/compound 2 (3.27 g, 10.0 mmol, 1.04 eq) was introduced to the stirring solution of VA. The reaction mixture was stirred overnight at room temperature and subsequently diluted with chloroform (300 mL), washed with ice-cold water (200 mL) and brine (15 mL), twice. The aqueous layer was back-extracted with 30 mL of chloroform and the combined organic layers were washed with 100 mL of water and 10 mL of brine. The organic layer was evaporated under reduced pressure to ca. 15 mL and precipitated into 1:1 (v/v) diethyl ether/pentane, and vortexed and centrifuged. SVA was obtained as a white pellet which was dried under in-house vacuum for 24 h to yield 2.81 g of SVA (73.1 % yield).

Synthesis of SVA-PAB-OH (Compound 4)

SVA (2.48g, 6.0 mmol, 1 eq) and PABA (0.88g, 7.2 mmol, 1.2 eq) were added into a round bottom flask and stirred in 50 mL DCM/MeOH (4:1) for 10 minutes. To the stirring solution, EEDQ (2.97g, 12.0 mmol, 2 eq) was slowly added over 5 minutes. The reaction mixture was allowed to stir overnight at room temperature. The reaction mixture was diluted with

chloroform (100 mL) and extracted with water (70 mL) and brine (10 mL), twice. The aqueous layers were back-extracted with chloroform (30 mL). The combined organic layers were subsequently extracted with water and brine. The organic layer was concentrated, and the resultant viscous liquid was purified by silica gel column chromatography running 95:5 (v/v) chloroform:methanol. The SVA-PABA fractions (Rf = 0.6) were collected and precipitated into 1:1 (v/v) diethyl ether/pentane. The precipitate was vortexed and centrifuged and the pellet was dried under in-house vacuum overnight.

Synthesis of SVA-PAB-PNP (Compound 5)

SVA-PAB-OH (1.82 g, 3.6 mmol, 1 eq) and PNP-Cl (1.524 g, 7.56 mmol, 2.1 eq) were dissolved in 80 mL of THF. The solution was cooled in an ice bath before the addition of TEA (801.4 mg, 7.92 mmol, 2.2 eq). The mixture was stirred on ice for 30 minutes, and allowed to warm up to room temperature on its own accord for a total reaction time of 24 hours. The resultant reaction mixture was filtered to remove the TEA salt. The filtrate was concentrated to a thick yellow oil via rotary evaporation, precipitated into diethyl ether, vortexed and centrifuged. The pellet was collected by decanting the ether layer, taken up in 150 mL chloroform and washed with 100 mL water and 15 mL brine, respectively. The organic phase was dried over anhydrous sodium sulfate, before concentration and final precipitation in 1:1 (v/v) diethyl ether/pentane. Following centrifugation, the pellet was dried under in-house vacuum to yield 1.74 g of SVA-PAB-PNP (72.5% yield)

Synthesis of STING monomer (SVA-PAB-STING) (Compound 6)

To synthesize the final STING agonist prodrug monomer, STING Agonist-3 (280.8 g, 0.375 mmol, 1 eq) and SVA-PAB-PNP (301.8 mg, 0.45 mmol, 1.5 eq) were dissolved in 8.3 mL of anhydrous DMF (stored under inert atmosphere). The solution was sparged with argon, and cooled on an ice bath before the addition of DIPEA (242.3 mg, 1.875 mmol, 5 eq) and DMAP (45.8 mg, 0.46 mmol, 1.22 eq). The mixture was stirred on ice for 30 minutes, and then at room temperature for 24 hours. Thereafter, the mixture was diluted with 3:1 (v/v) CHCl3:iPrOH (35 mL) and washed three times with cold 2:1 (v/v) saturated NH4Cl:water (19 mL each time). The aqueous wash was back-extracted with 3:1 (v/v) CHCl3:iPrOH (10 mL). The combined organic layers were dried with anhydrous sodium sulfate. The organic phase was concentrated to a thick oil with high-vacuum rotary evaporation with a dry ice cold finger, and combined with 60A silica gel for dry column loading. The monomer was purified using a CombiFlash NextGen 300 flash chromatography system running a binary dichloromethane and 10:1 (v/v) MeOH:NH4OH mobile phase (0-15% MeOH:NH4OH gradient over 15-20 minutes), collecting the second peak (Rf = 0.5 with 18% MeOH:NH4OH in DCM). The fractions were combined and subject to rotary evaporation to near-dryness (a thick gel) and completely evaporated with in-house vacuum for 2-3 days to yield 150-200 mg (30-40% yield) of SVA-PAB-STING.

It is noted that manual gradient column chromatography can also be employed (5-12% MeOH:NH4OH gradient with 2% increments every 100 mL mobile phase) with some co-eluted fractions. Running the reaction for extended amounts of time (72 h) decreased yield (around 50 mg, 10% yield).

For characterization, mass spectra were recorded on a Bruker EsquireLC Ion Trap (Bruker Corporation, Billerica, MA) Electrospray Ionization Mass Spectrometer instrument with direct injection in methanol (20 μg/mL) in negative ion mode. ^{1}H NMR spectra were recorded on a Bruker NEO 500 (Bruker Corporation, Billerica, MA) autosampler nuclear magnetic resonance (NMR) instrument in deuterated DMSO (DMSO-d6)

^{1}H NMR (500 MHz, DMSO-d6) δ 12.81 (s, 2H), 9.90 (s, 1H), 8.15 (d, J = 7.0 Hz, 1H), 8.01 – 7.90 (m, 3H), 7.71 (dd, J = 8.4, 1.7 Hz, 1H), 7.66 (s, 1H), 7.62 – 7.56 (m, 2H), 7.41 (d, J = 8.4 Hz, 1H), 7.34 – 7.27 (m, 4H), 6.53 (d, J = 10.3 Hz, 2H), 6.03 (s, 1H), 5.98 (dd, J = 13.3, 7.8 Hz, 1H), 5.76 – 5.66 (m, 2H), 5.01 (s, 2H), 4.94 (d, J = 5.3 Hz, 2H), 4.81 (d, J = 5.7 Hz, 2H), 4.52 (d, J = 6.9 Hz, 4H), 4.38 (p, J = 7.1 Hz, 1H), 4.26 (dt, J = 8.0, 4.1 Hz, 4H), 4.20 – 4.13 (m, 3H), 4.09 (t, J = 6.2 Hz, 2H), 3.78 (p, J = 6.1 Hz, 1H), 2.11 (d, J = 6.1 Hz, 5H), 1.97 (dt, J = 12.1, 6.4 Hz, 3H), 1.87 (d, J = 1.3 Hz, 3H), 1.33 – 1.23 (m, 9H), 1.05 (d, J = 6.1 Hz, 5H), 0.85 (dd, J = 17.6, 6.8 Hz, 6H), -0.06 (s, 1H).

Polymer synthesis

ManEMA (200 mg, 0.68 mmol, 35.7 eq), SVA-PAB-STING (55.9 mg, 0.044 mmol, 2.2 eq), were all dissolved in DMSO-d6 (0.34 mL) in a 5 mL oven-dried pear-shaped flask. Trioxane (15 mg) was added as an internal standard for monomer conversion calculations. Next, a stock solution (20.2 mg/mL in DMSO-d6) of the RAFT chain transfer agent ECT (0.25

mL, 5.05 mg, 0.019 mmol, 1 eq) was added. Thereafter, a stock solution of V70 (17.7 mg/mL in anhydrous DMF) was prepared and quickly added to the solution (0.05 mL, 0.88 mg, 0.0029 mmol, 0.15 eq). After taking a small aliquot (0.02 mL) for NMR conversion analysis, the reaction vessel was sealed with a septa and purged with argon gas for 30 minutes. The flask was then placed into an oil bath set to 42 C, and allowed to react for 22 hours. Upon completion, the reaction mixture was dialyzed against DMSO (500 mL) for 3 days using a SnakeSkin 3.5 kDa MWCO regenerated cellulose membrane, changing the DMSO twice daily. The reaction mixture was then dialyzed against ice-cold water (4C, 4000 mL) in a cold room for 2 days to remove DMSO, changing water twice daily. The dialysate was then lyophilized for 3 days to yield 188 mg (85% gravimetric conversion) of polySTING.

For the synthesis of rhodamine-labeled polySTING (polySTING-Rh), 3.8 mg of RhMA (0.0055 mmol, 0.38 eq) was added to the following: 0.48 mL DMSO-d6, 150 mg of ManEMA (0.51 mmol, 35.3 eq), 42.4 mg of SVA-PAB-STING (0.038 mmol, 2.3 eq), 3.82 mg of ECT (0.015 mmol, 1 eq) and 0.67 mg of V70 (0.0022 mmol, 0.15 eq). Following the addition of RhMA, the above protocol was exactly repeated.

For the synthesis of rhodamine-labeled non-glycan STING drugamer control (MSEMA-STING), 5.7 mg of RhMA (0.0084 mmol) was added to the following: 0.48 mL DMSO-d6, 140 mg of MSEMA (0.79 mmol, 42.3 eq), 54.1 mg of SVA-PAB-STING (0.042 mmol, 2.3 eq), 4.9 mg ECT (0.018 mmol, 1 eq) and 0.87 mg of V70 (0.0028 mmol, 0.15 eq). Following the addition of RhMA, the above protocol was exactly repeated.

2. Characterization and Experimental Methods

Polymer Characterization

All ^{1}H NMR spectra were recorded on a Bruker NEO 500 (Bruker Corporation, Billerica, MA) autosampler nuclear magnetic resonance (NMR) instrument in deuterated DMSO (DMSO-d6). Drug incorporation into polymers was assessed by ^{1}H NMR analysis of a known polymer mass (typically >10mg in 600 uL solvent) and the internal standard levofloxacin (typically 1 mg/mL in solvent) (Levofloxacin δ 8.9 (s, 1H), STING δ 6.45 (d, 2H)). Polymer molecular weights (Mw, Mn) and polydispersity index (Đ = Mw/Mn) were determined by gel permeation chromatography (GPC). The system consisted of an Agilent 1260 HPLC stack running heated LiBr-supplemented (0.1% w/v) DMF mobile phase at a flow rate of 1 mL min^{-1} through a semi-prep three-column setup (Tosoh TSKGel alpha-4000 to Tosoh TSKGel alpha-300 to Phenomenex Phenogel 10^3 A). Samples (10 mg/mL) were filtered through a 0.2 µm hydrophilic PTFE filter before analysis. The unimeric form of the polymer in solution was verified by Dynamic Light Scattering (DLS) on a DynaPro NanoStar (Wyatt Technology, Santa Barbara, CA) through different polymer concentrations (200-1.25 mg/mL) in PBS.

Formulation of STING treatments

For the free drug STING-3, the received powder was dissolved into sterile DMSO at 4 mg/mL with sonication, and stored at -20C until use, where it is diluted to *in vivo* concentrations with 1X DPBS. For the polymeric STING prodrug polySTING, the lyophilized powder was reconstituted in 1X DPBS at *in vivo* concentrations. All formulations were sterile-filtered through a 0.2 µm PVDF membrane before use.

Cell lines and animals

The B16F10 cell line was cultured in DMEM supplemented with 10% FBS and 1% P/S. The 4T1 cell line was cultured in RPMI supplemented with 10% FBS and 1% P/S. Cells were incubated at 37°C and 5% CO_2. Female C57BL/6 mice and female BALB/c mice aged 6 to 8 weeks were purchased from Charles River Laboratories and The Jackson Laboratories. All animal studies were approved by the University of Washington Institutional Animal Care and Use Committee (IACUC).

Bone marrow-derived macrophage polarization

Bone marrow cells were extracted from murine femurs and tibias. Cells were seeded in 10 cm non-TC plates with plating media: DMEM + 10% FBS + 1% P/S + 20 ng/ml M-CSF. On day 4, 10 more ml of plating media was added per plate. On day 7, cells were polarized to either M1 using 100 ng/ml LPS + 20 ng/ml IFNγ, or M2 using 20 ng/ml IL-4. After a 48-hour incubation, M1 cells were treated with PBS and M2 cells were treated with either PBS, free STING, or polySTING at a concentration of 20 µg/ml. After a 24 hour incubation, cells were stained for viability with Zombie NIR, blocked with anti-

CD16/32, and finally stained with anti-CD206-PerCP-eFluor710, anti-CD11b-FITC, anti-CD163-PE, and anti-CD86-BV510. An Attune NxT flow cytometer was used to analyze the data. M2 BMDMs were treated with PBS, free STING, or polySTING at a concentration of 20 μg/ml for 48 hours. RNA was extracted using RNeasy mini kit (Qiagen). cDNA was synthesized using the high-capacity cDNA reverse transcription kit (Applied Biosystems). qPCR was performed to analyze the amount of cDNA. Arg1 and Nos2 expressions were normalized to the GAPDH housekeeping gene. The DNA primers were used as follows: GAPDH: AATGGATTTGGACGCATTGGT; TTTGCACTGGTACGTGTTGAT. ARG1: CAAGACAGGGCTCCTTTCAG; TGGCTTATGGTTACCCTCCC. NOS2: GATGTTGAACTATGTCCTATCTCC; GAACACCACTTTCACCAAGAC.

Toxicity study

Female BALB/c mice were injected with free STING at 30 μg and polySTING at 10, 20 and 30 μg (STING equivalent) on days 0 and 4, and were observed for signs of decrease in activity, hunched posture and weight loss. Liver toxicity was analyzed by injecting PBS, free STING and polySTING at 10 μg through the tail vein twice, with 3 days apart to C57BL/6 mice and collecting serum 24 h post 2nd injection. Serum alanine aminotransferase (ALT) level was measured using an ALT activity assay (Sigma). Aspartate Aminotransferase (AST) level was taken from frozen serum using an AST activity assay kit (Abcam). A Tecan Infinite plate reader was used to take the colorimetric readouts.

ELISA for cytokine quantification

Female C57BL/6 mice were injected with 30 μg free drug STING agonist-3 or polySTING containing 10, 20 or 30 μg of STING agonist-3 through the tail vein. Blood was collected through tail vein 4 h post injection in EDTA-treated tubes. Blood was spun down for 10 min at 1000 g and upper layer plasma was collected for ELISA to measure IFNβ expression level (Invivogen).

Interferon-stimulated gene expression

Female C57BL/6 mice with B16F10 tumors around 100-200 mm^3 were injected with PBS or 10 μg free STING agonist-3 or 10 μg polySTING through the tail vein. Tumors and TDLNs were collected 4 hr post injection. RNA was extracted from tumor tissues or TDLN cells using RNeasy mini kit (Qiagen). cDNA was synthesized using the high-capacity cDNA reverse transcription kit (Applied Biosystems). qPCR was performed to analyze the amount of cDNA. Ifnb1 and cxcl10 expressions were normalized to the GAPDH housekeeping gene. The DNA primers were used as follows: GAPDH: AATGGATTTGGACGCATTGGT; TTTGCACTGGTACGTGTTGAT. Ifnb1: AGCTCCAAGAAAGGACGAACA; GCCCTGTAGGTGAGGTTGAT. Cxcl10: CCAAGTGCTGCCGTCATTTTC; GGCTCGCAGGGATGATTTCAA.

Tissue processing

Tumors were cut into small pieces, treated with 20 U/mL type IV collagenase + 125 U/mL DNase I, and dissociated with the GentleMACS Dissociator. Tumors were then incubated on a rotating platform at 37 °C for 30 min. Single-cell suspensions were obtained by passing homogenate through a 70 μm cell strainer. Cells were treated with the ACK lysing buffer to remove red blood cells. Inguinal lymph nodes were dissociated with pipette tips and digested with 50 U/mL type IV collagenase + 100 U/mL DNase I. Cells were incubated at 37 °C for 30 min with gentle agitation. Single-cell suspensions were obtained by passing homogenate through a 70 μm cell strainer.

Pharmacokinetic characterization

Female C57BL/6 mice were inoculated with 2 × 105 B16F10 cells in the right-hind flank on day -8. On day 0 (tumor volume approx. 100-200 mm3), they were treated with polySTING (10 μg STING equivalent) through tail-vein injection. 30 minutes post-administration, mice were sacrificed and whole blood collected through cardiac puncture, followed by tumor isolation. Tumor and plasma were snap frozen in dry ice and stored at -80oC until analysis. Samples were processed and analyzed with tandem LC/MS-MS (Xevo TQ-S, Waters Corporation, Milford, MA) according to previously published procedures[1]. The MS instrument was operated on a positive ion multiple-reaction monitoring (MRM) mode monitoring 136 m/z for the

STING agonist and 112 m/z for the internal standard gemcitabine (LLOQ-ULOQ 0.57-1248 ng/mL plasma or 2.57-624.0 ng/g tumor). The LC was performed with an InfinityLab Proshell 120 Aq-C18 analytical (2.1x150mm) column (Agilent, Santa Clara, CA). The LC solvents were water supplemented with 0.1% (v/v) formic acid (solvent A), and acetonitrile supplemented with 0.1% (v/v) formic acid (solvent B). The linear gradient used was from 95:5 A:B to 0:100 A:B over 8 minutes at a 0.25 mL/min flow rate.

Tumor cellular uptake studies

Female C57BL/6 mice were inoculated with 2×10^5 B16F10 cells in the right-hind flank on day -8. On day 0 (tumor volume approx. 100-200 mm3), they were treated with either MSEMA-STING-Rh or polySTING-Rh (10 μg STING equivalent) through tail-vein injection. 30 minutes post-administration, tumors were harvested and made into single-cell suspension as described previously. Cells were stained for viability with Zombie Violet viability kit, blocked with Trustain anti-mouse CD16/32, and then stained with anti-CD45-APC/Cy7, anti-CD11c-FITC or anti-F4/80-FITC. An Attune NxT flow cytometer was used to analyze the data.

Organ-level biodistribution studies

Female C57BL/6 mice were inoculated with 2 × 105 B16F10 cells in the right-hind flank on day -8. On day 0 (tumor volume approx. 100-200 mm3), they were treated with polySTING-Rh (10 μg STING equivalent) through tail-vein injection. 30 minutes post-administration, mice were sacrificed and perfused for organ collection. Tissues were homogenized in RIPA buffer with 2500 U/mL DNase-I and supernatant were collected after centrifugation. The fluorescent polySTING-Rh signal of organ homogenates was measured on a Tecan Infinite M200 pro plate reader (Tecan Life Sciences, Mannedorf, Switzerland, Ex/Em: 530/580 nm). Homogenate protein level was measured using the Pierce BCA protein assay kit. Calibrators were prepared by spiking naive organ homogenates with stock solutions of polySTING-Rh covering a final concentration range of 0.85-6.79 μg/mL homogenate of polySTING-Rh.

Tumor-infiltrating lymphocytes (TIL) and TDLN analysis

Female C57BL/6 mice were inoculated with 2×10^5 B16F10 cells in the right-hind flank on day -8. On days 0 (tumor volume approx. 100-200 mm3) and 4, they were treated with either PBS or polySTING (10 μg STING equivalent) through tail-vein injections. On day 5, tumors and the tumor-draining lymph nodes (TDLNs, inguinal LN in the same flank as tumor) were harvested. Tissues were made into single-cell suspension as previously described.

Tumor cells were stained for viability with Zombie Violet viability kit, blocked with Trustain anti-mouse CD16/32, and then stained in three plates for T cells (anti-CD45-APC/Cy7, anti-CD3-PE/Cy5, anti-CD4-PE, anti-CD8-FITC), macrophages (anti-CD45-APC/Cy7, anti-F4/80-FITC, anti-CD80-PE, anti-CD206-BV605), and DCs (anti-CD45-APC/Cy7, anti-CD11c-FITC, anti-CD80-PE, anti-MHCII-PE/Cy5). In a separate study, tumor cells were stained for viability with Zombie NIR viability kit, blocked with Trustain anti-mouse CD16/32, and then stained in two plates for DCs (anti-CD11c-APC, anti-CD8-FITC, anti-CD103-PE, anti-CD86-BV510) and T cells (anti-CD3-PE/Cy5, anti-CD8-FITC, anti-TCF1-PE, anti-PD1-eFluor450).

TDLN cells were stained for viability with Zombie NIR viability kit, blocked with anti-mouse CD16/32, and then stained in two plates for DCs (anti-CD11c-APC, anti-CD8-FITC, anti-CD103-PE, anti-CD86-BV510) and T cells (anti-CD3-eFluor450, anti-CD4-PE, anti-CD8-FITC). In a separate study with 30 μg STING agonists and a single tail-vein injection, TDLN cells were stained for viability with Zombie NIR viability kit, blocked with anti-mouse CD16/32, and then stained in with anti-CD11c-APC, anti-CD86-BV510, anti-CD40-FITC and anti-CD80-PE. An Attune NxT flow cytometer was used to analyze the data.

Therapeutic studies

For the B16F10 tumor model, female C57BL/6 mice were inoculated with 2×10^5 B16F10 cells in the right-hind flank on day -8. On days 0, 4, and 8, mice were treated intravenously (tail vein on days 0, 4, and retro-orbitally on day 8) with PBS,

or either free STING or polySTING with an equivalent STING agonist dose of 10 µg. For the 4T1 tumor model, female BALB/c mice were inoculated with 5×10^5 4T1 cells in the mammary fat pad on day -8. On days 0, 7, and 14, mice were treated intravenously (tail vein on days 0, 7, and retro-orbitally on day 14) with PBS, or either free STING or polySTING with an equivalent STING agonist dose of 10 µg. On days 1, 8, and 15, mice were injected with 100 µg of anti PD-1 ICB via intraperitoneal injection. Tumor length and width were then measured using a vernier caliper, and volume was subsequently calculated using the formula: Volume = $(\text{Width}^2 \times \text{Length}) \div 2$. Weight was measured the same time as tumor measurements. Mice were euthanized if total tumor volume exceeded 2000 $mm^3$for B16F10 tumors, and 1500 mm^3 for 4T1 tumors, visible discharge was observed on ulcers of the tumors, signs of mortality such as excessive lethargy, or the body weight loss exceeded 20%.

Statistical analysis

Statistical analysis was performed using the Graphpad Prism software. Unpaired t test was used to compare two groups. One-way ANOVA with post-hoc Tukey HSD test was used to compare more than two groups. Log-rank test was used for survival analysis. Mixed-effects analysis of two-way ANOVA with Tukey's multiple comparison was used to analyze tumor growth curves.

3. Supporting Figures and Tables

	**Measured Concentration**	
**Sample Type (unit)**	**Free STING/diABZI**	**PolySTING**
**Plasma (ng/mL)**	**1.57**	**130.41**
**Tumor (ng/g)**	**103.15**	**2978.72**

Table S1. LC/MS-MS based pharmacokinetic characterization of polySTING and free STING agonist 30 minutes after intravenous administration.

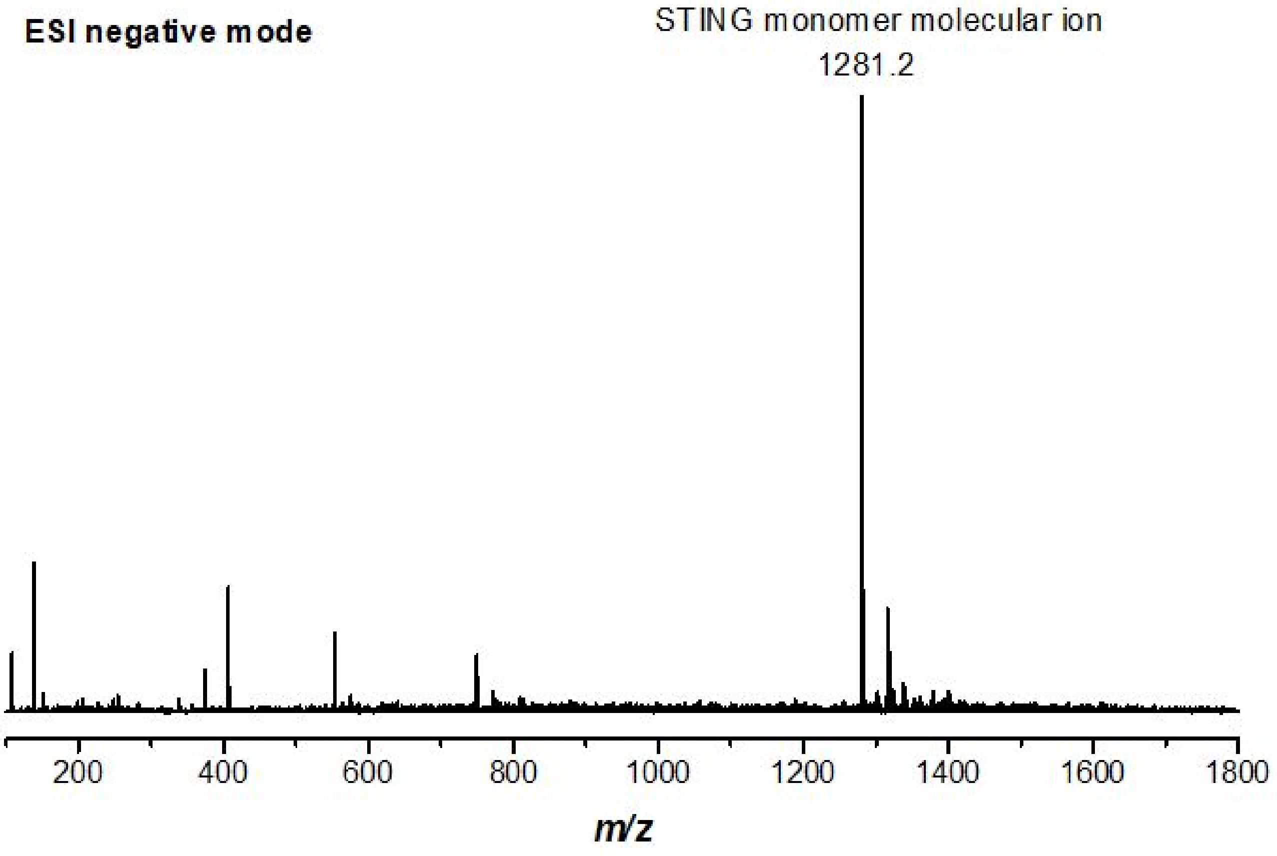

Figure S1. Mass spectrum of SVA-PAB-STING (Compound 6) in methanol.

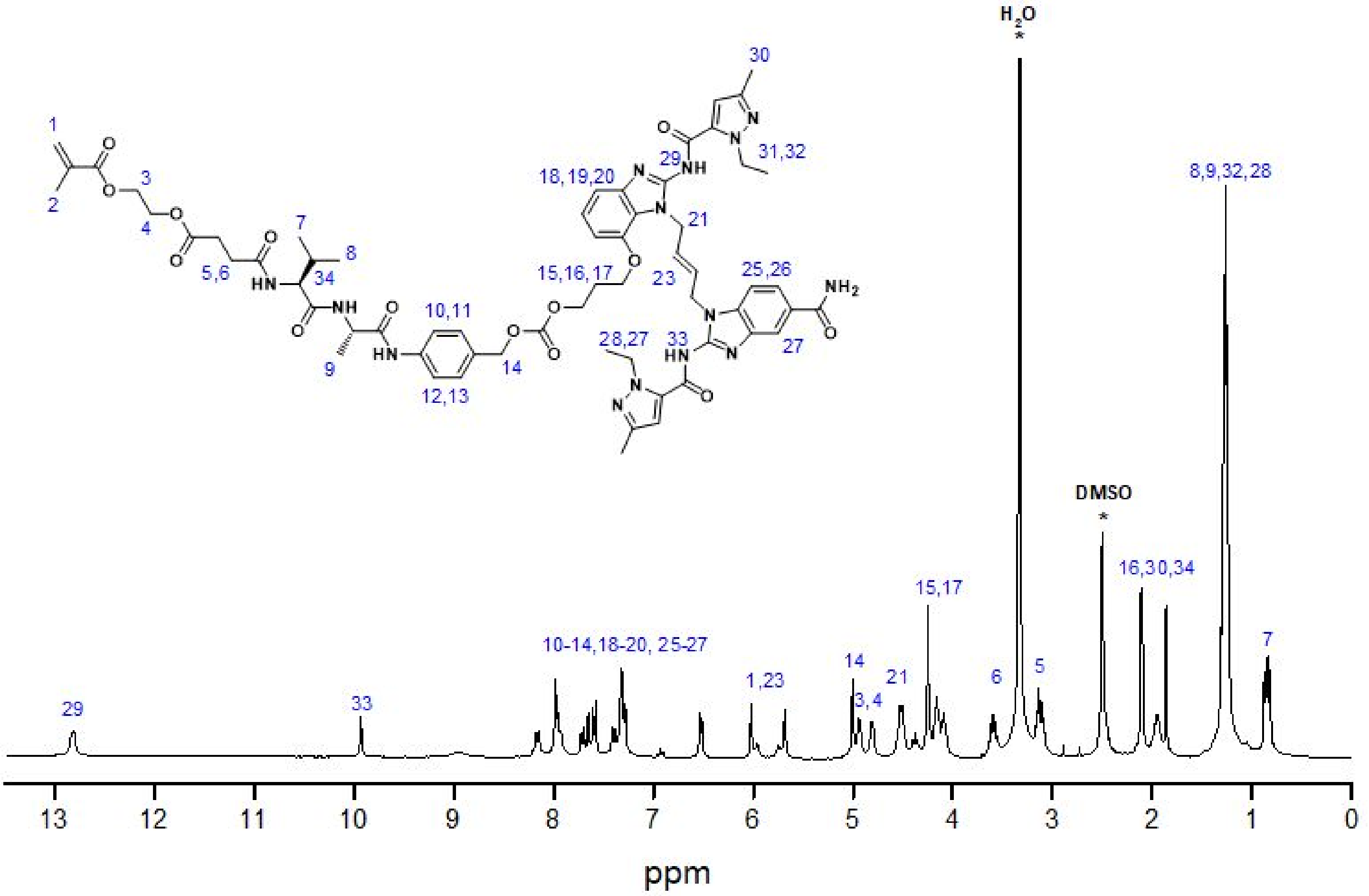

Figure S2. ^{1}H-NMR spectrum of SVA-PAB-STING (Compound 6) in DMSO-d6.

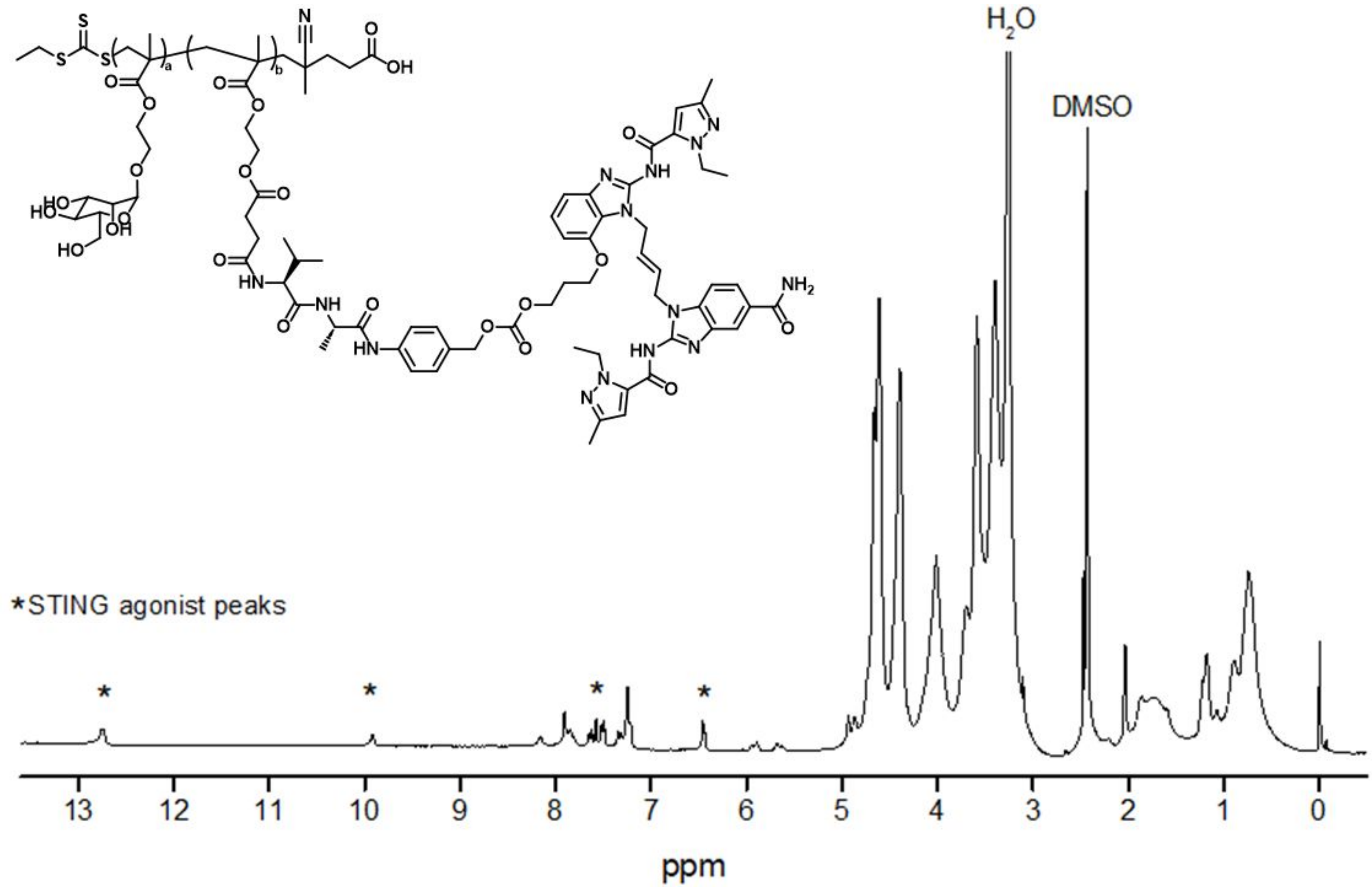

Figure S3. ^{1}H-NMR spectrum of polySTING in DMSO-d6.

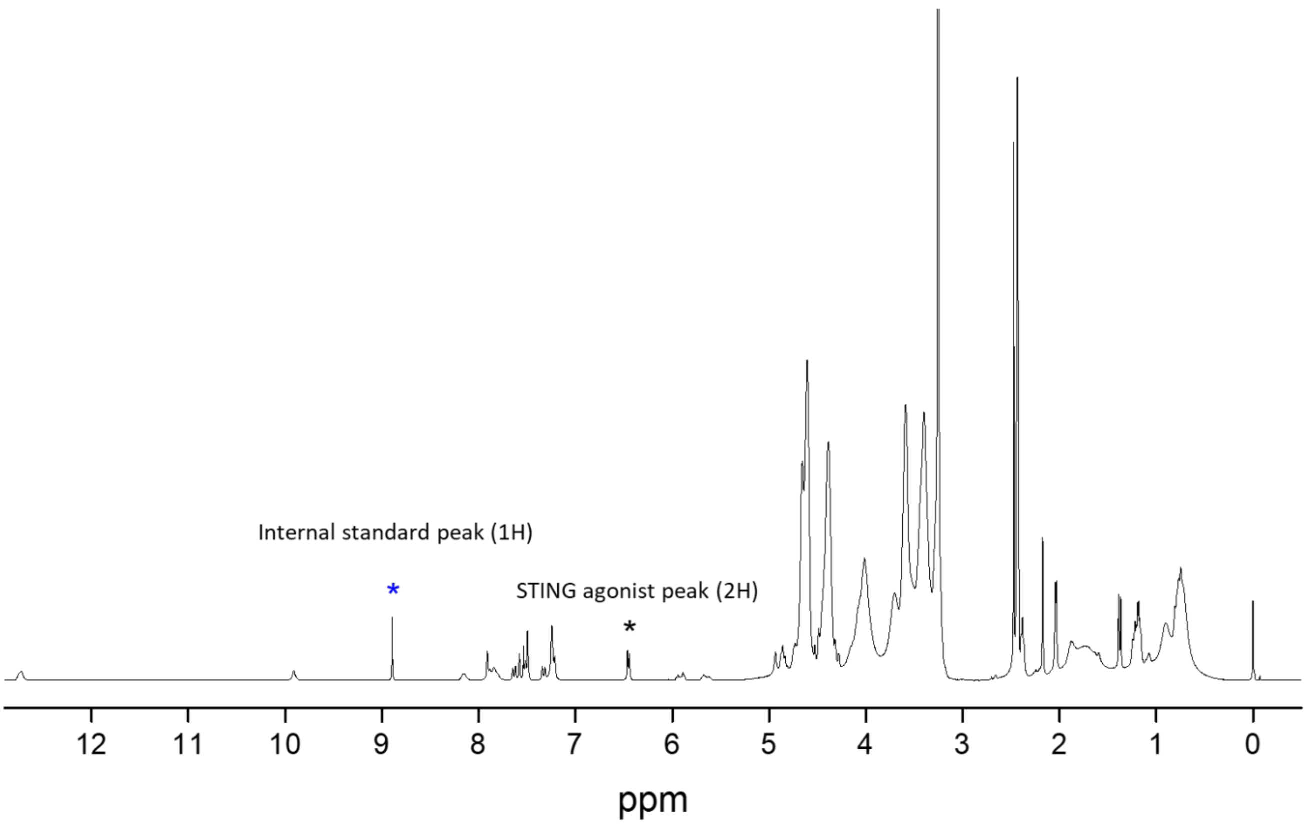

Figure S4. ^{1}H-NMR spectrum of polySTING and levofloxacin internal standard.

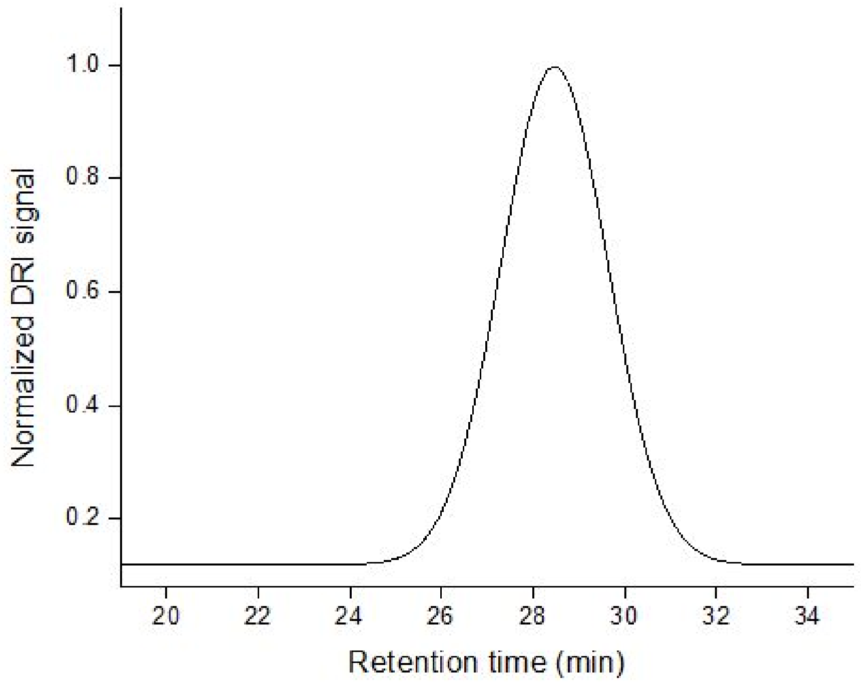

Figure S5. GPC spectrum of polySTING in supplemented DMF (1 g/L LiBr).

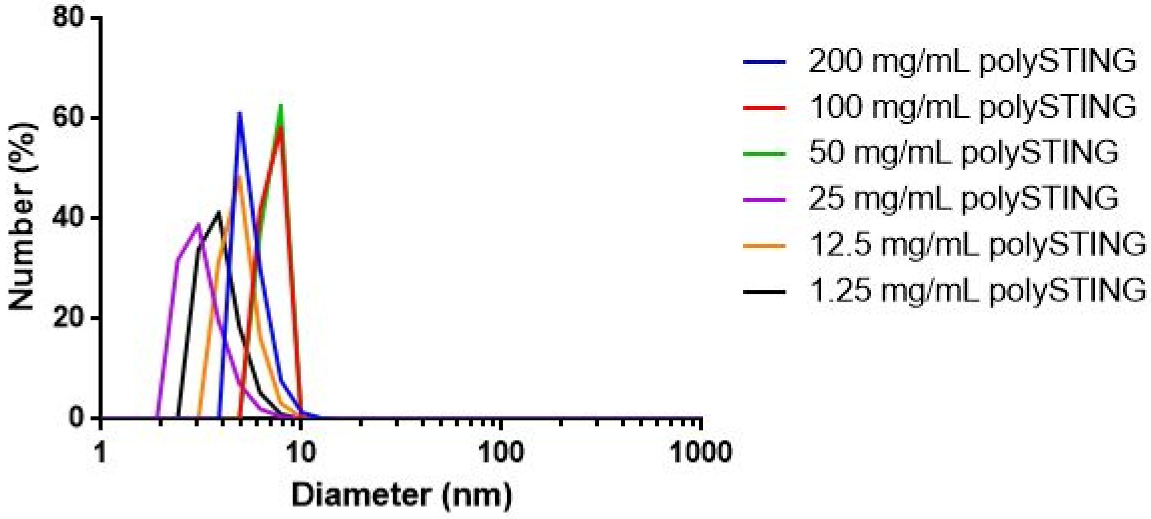

Figure S6. DLS characterization of polySTING in PBS at different concentrations.

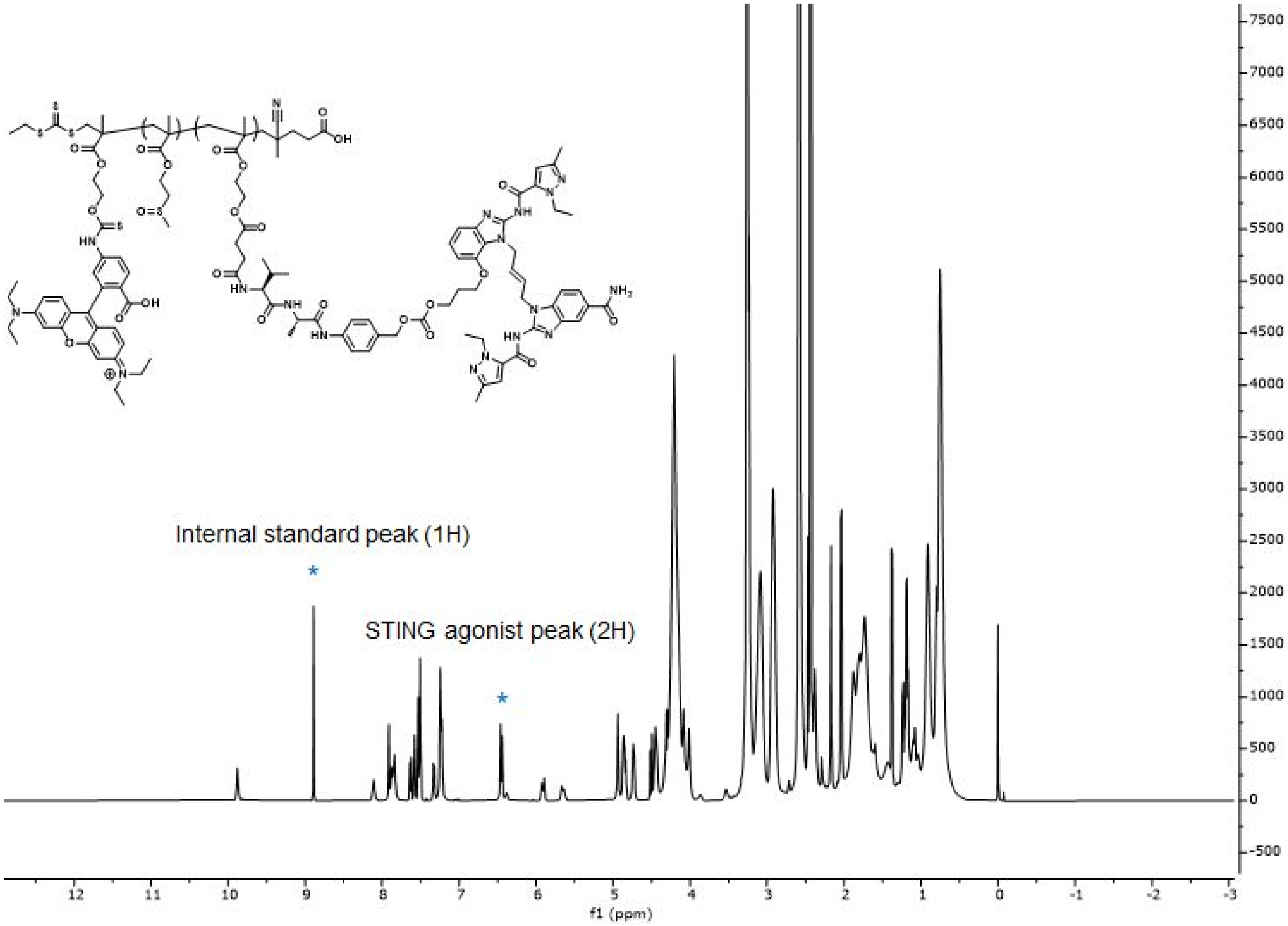

Figure S7. 1H-NMR of MSEMA-STING-Rh in DMSO-d6 with levofloxacin internal standard.

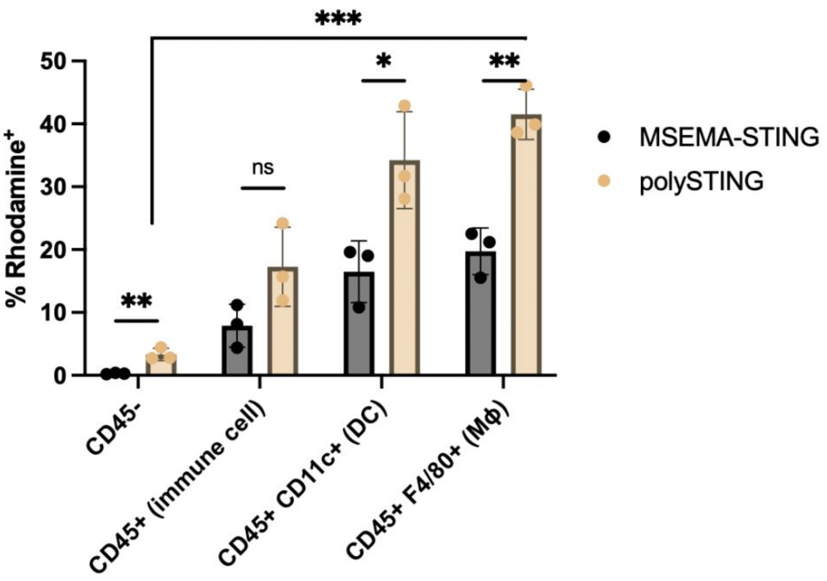

Figure S8. Rhodamine-labeled polySTING and MSEMA-STING uptake in B16F10 tumors 4 h post tail vein injection. Unpaired t-test was used to compare polySTING and MSEMA-STING under different cell subsets. One-way ANOVA with post-hoc Tukey's test was used to polySTING uptake in different cell types. (*p ≤ 0.05, **p ≤ 0.01, ***p ≤ 0.001, ns: not significant).

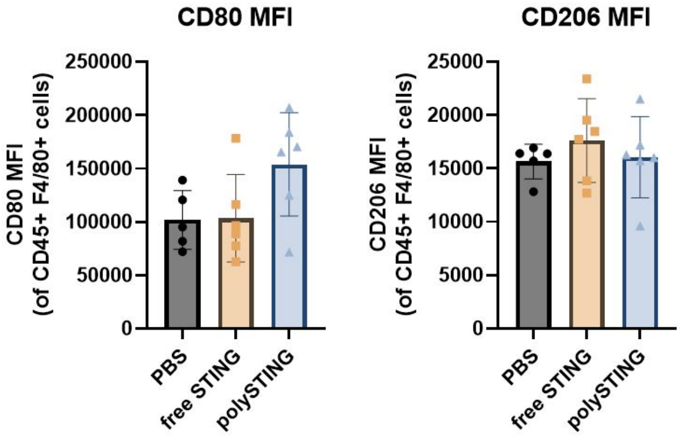

Figure S9. *In vivo* tumor-associated macrophage polarization.

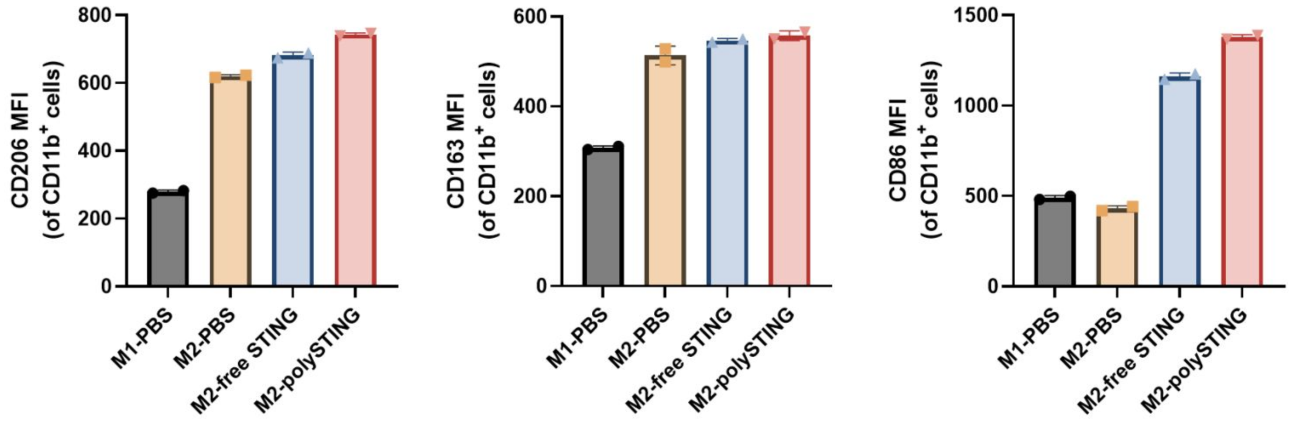

Figure S10. *In vitro* bone marrow-derived macrophage (BMDM) polarization via flow cytometry.

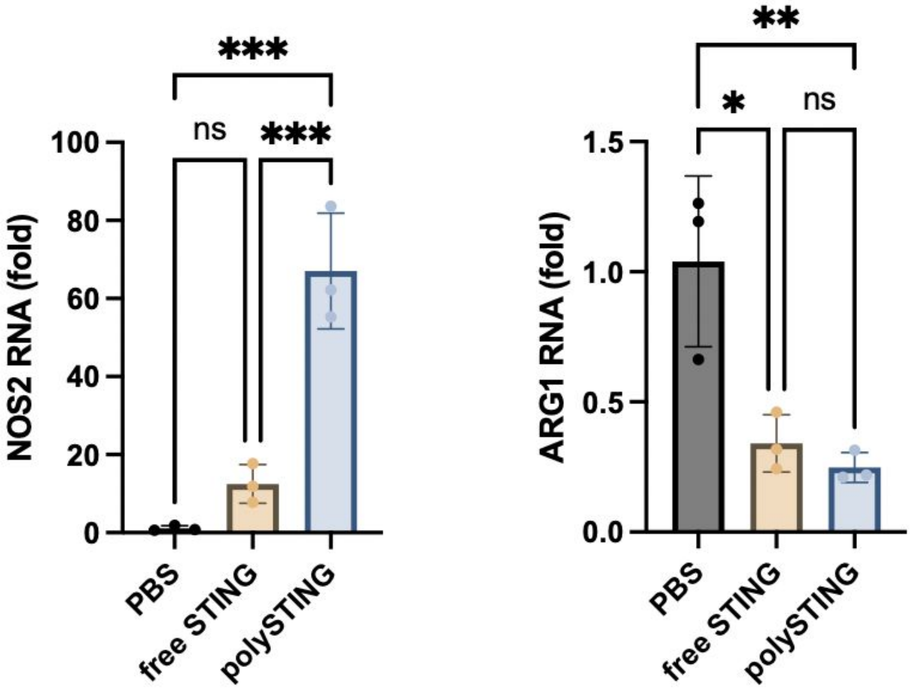

Figure S11. RT-qPCR of BMDM polarization genes in vitro.

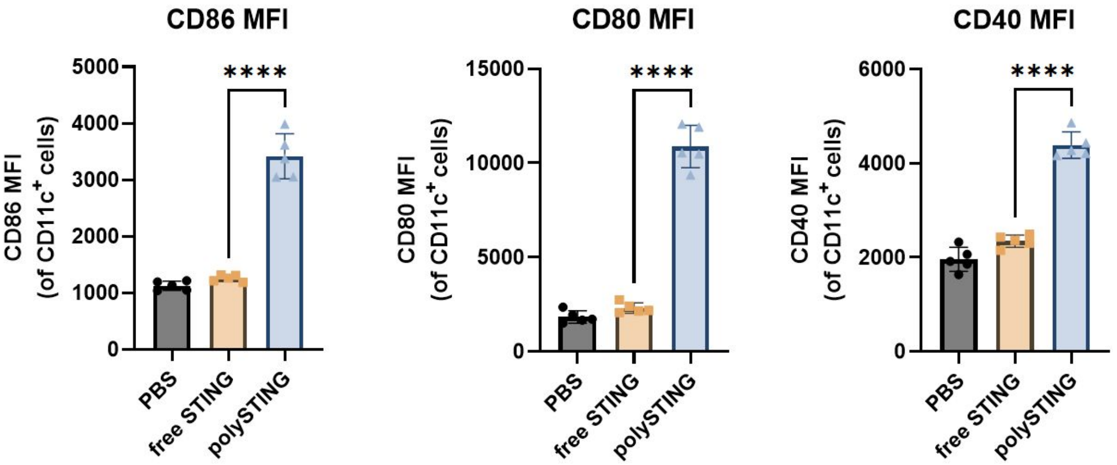

Figure S12. *In vivo* DC maturation in TDLNs

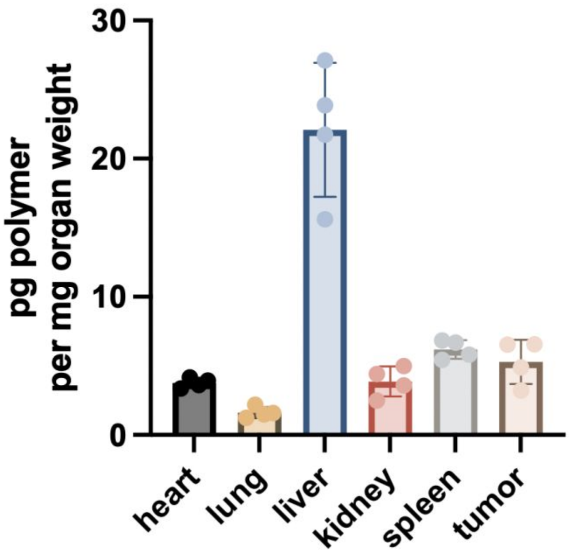

Figure S13. Systemic biodistribution of polySTING-Rh as measured by organ homogenate fluorescence, calibrated against polySTING-Rh-spiked naive homogenate.

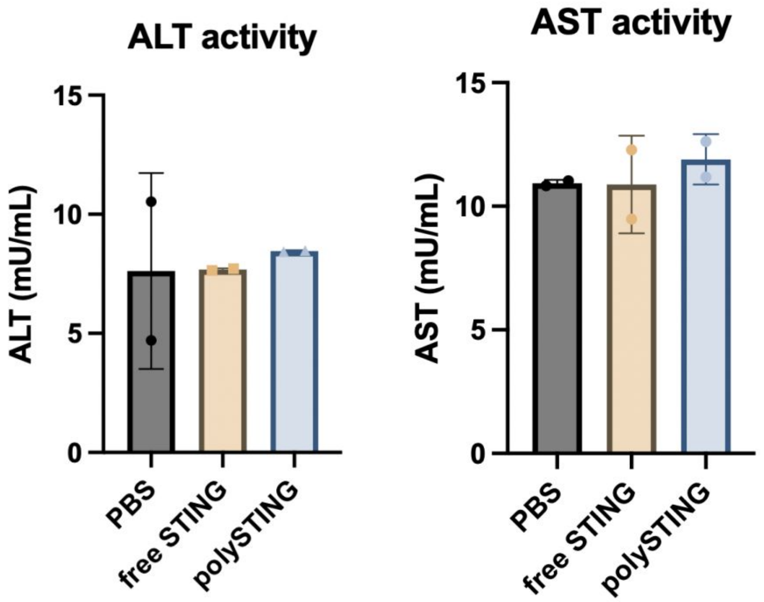

Figure S14. Liver toxicity assay of polySTING, as measured by serum alanine aminotransferase (ALT) and aspartate aminotransferase (AST) levels.

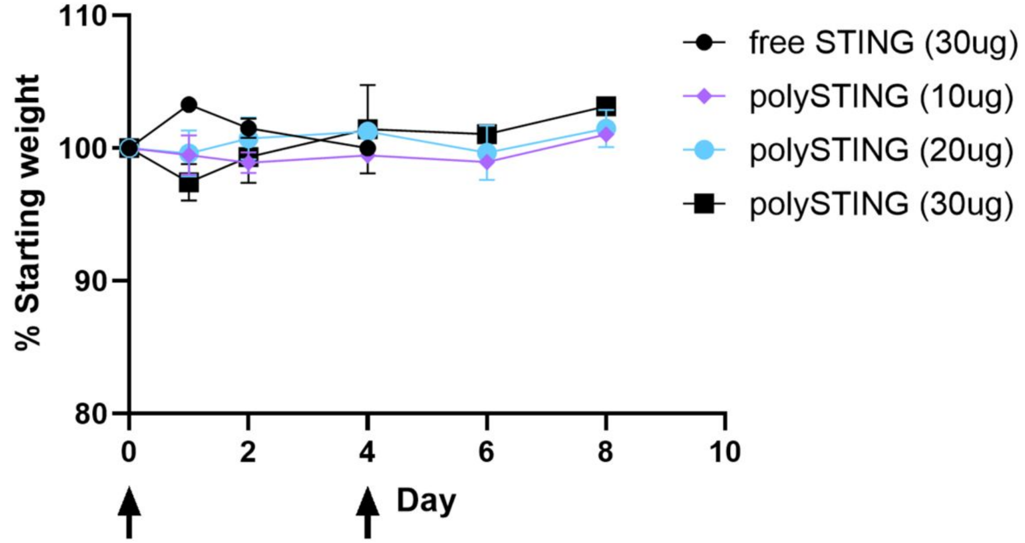

Figure S15. Weight loss in non-tumor bearing mice.

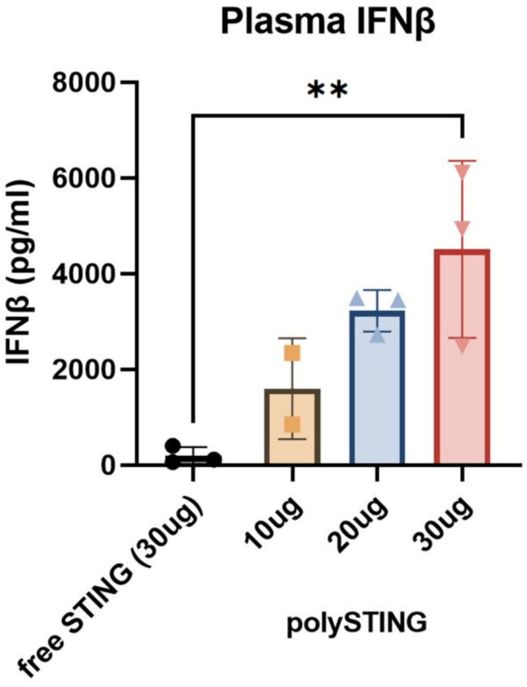

Figure S16. Plasma IFNβ level in healthy C57BL/6 mice 4 h post treatment with free STING and polySTING at different doses (N=3).

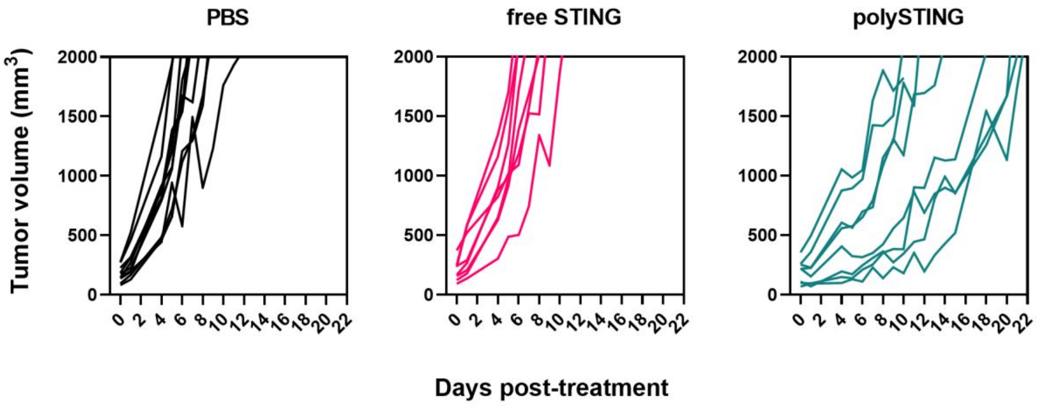

Figure S17. Individual tumor growth curves for B16F10 tumor reduction study.

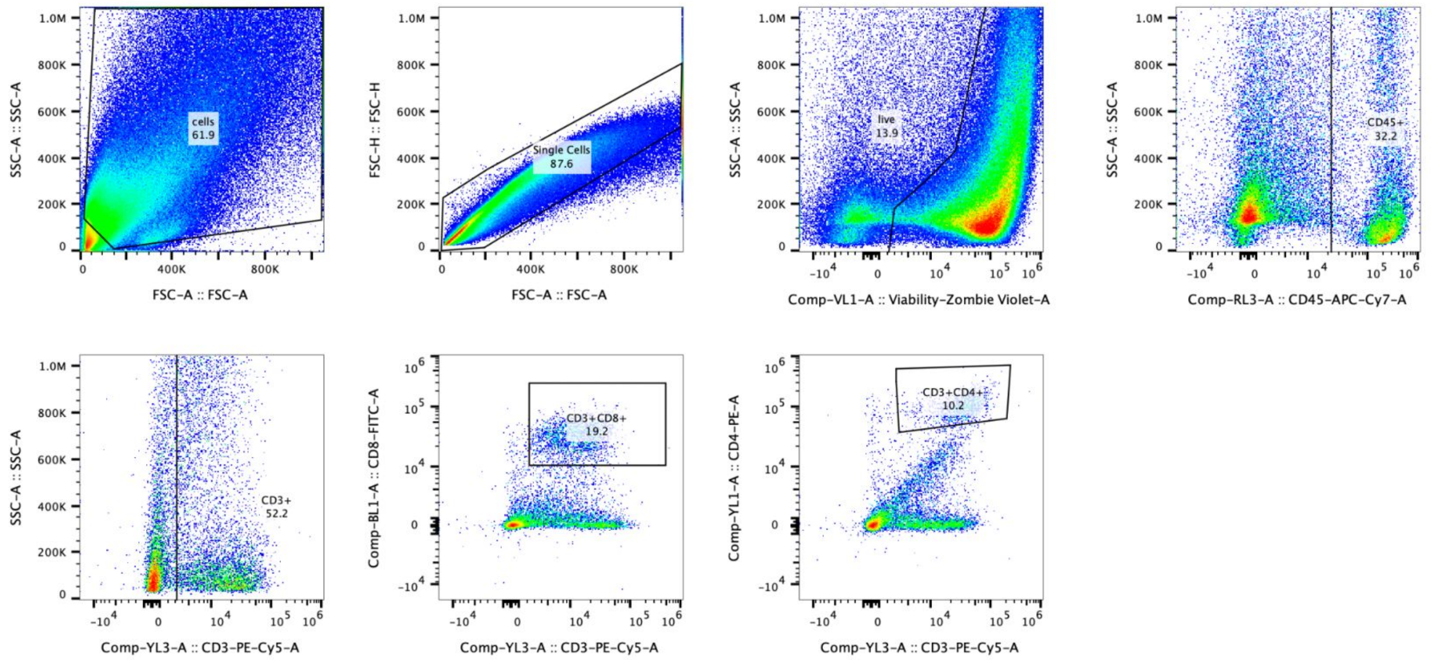

Figure S18. Gating on tumor-infiltrating CD4^{+} and CD8^{+} T cells.

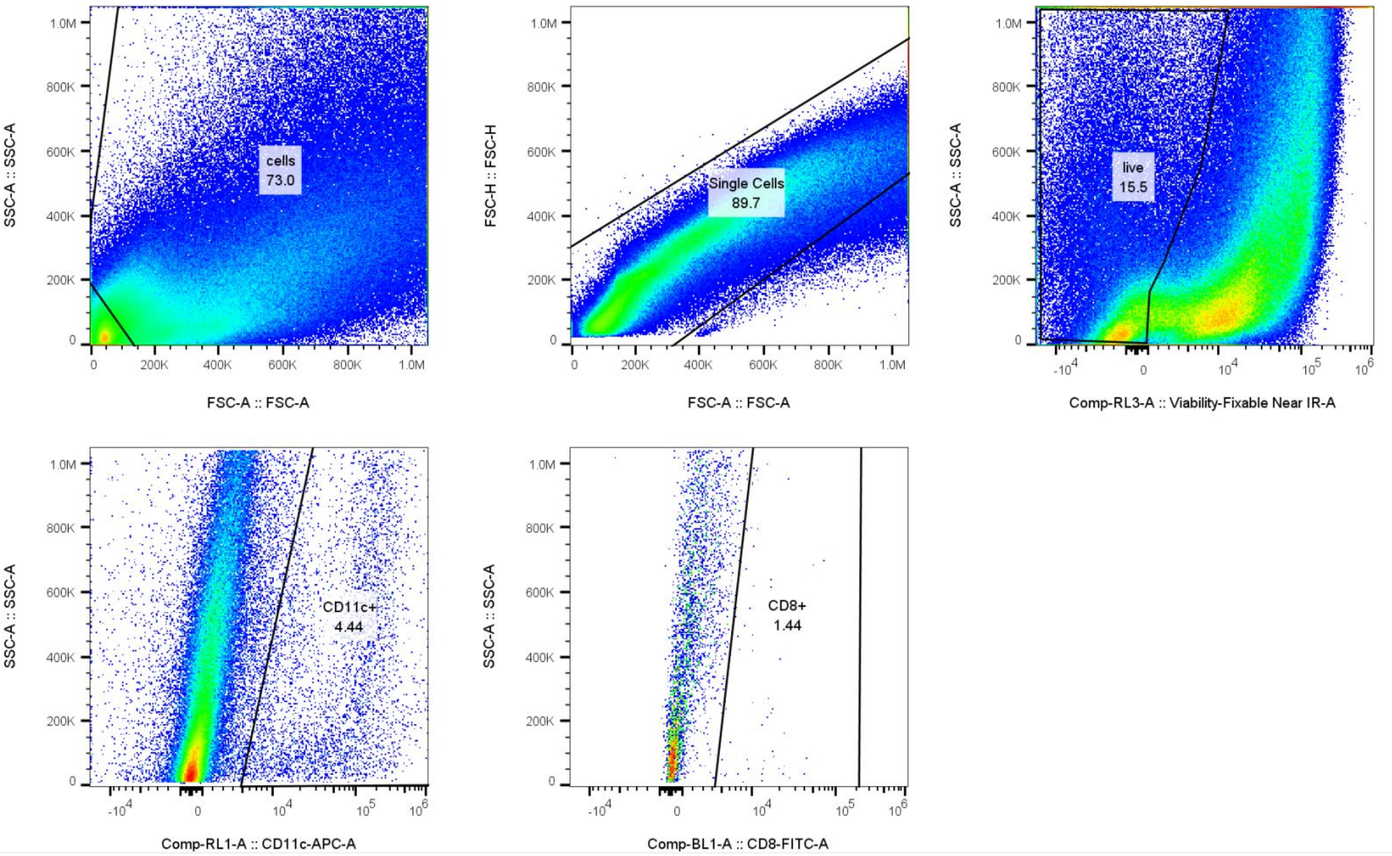

Figure S19. Gating on CD8^{+} CD11c^{+} DCs in tumors.

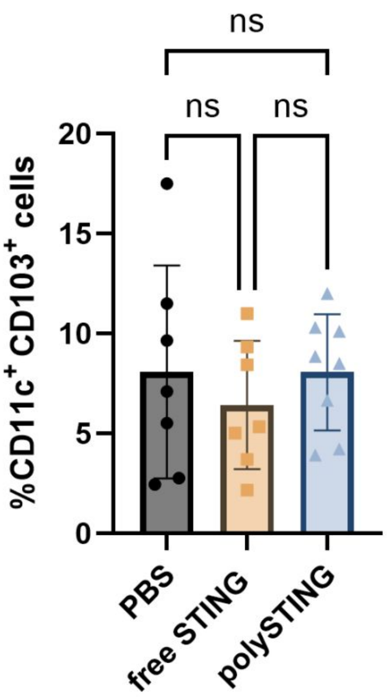

Figure S20. Assessment of $CD11c^{+}$ $CD103^{+}$ DCs in the TME

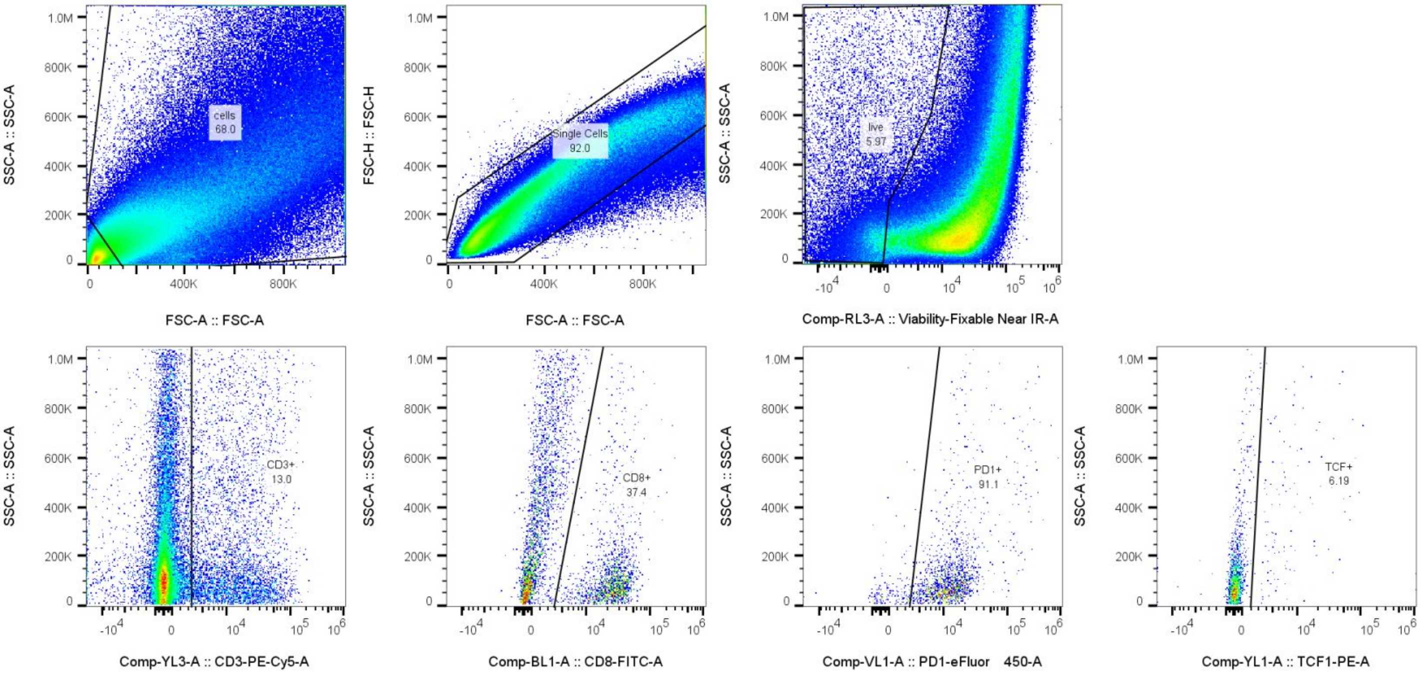

Figure S21. Gating on TCF-1^{+}, PD-1^{+} $CD8^{+}$ T cells in tumors.

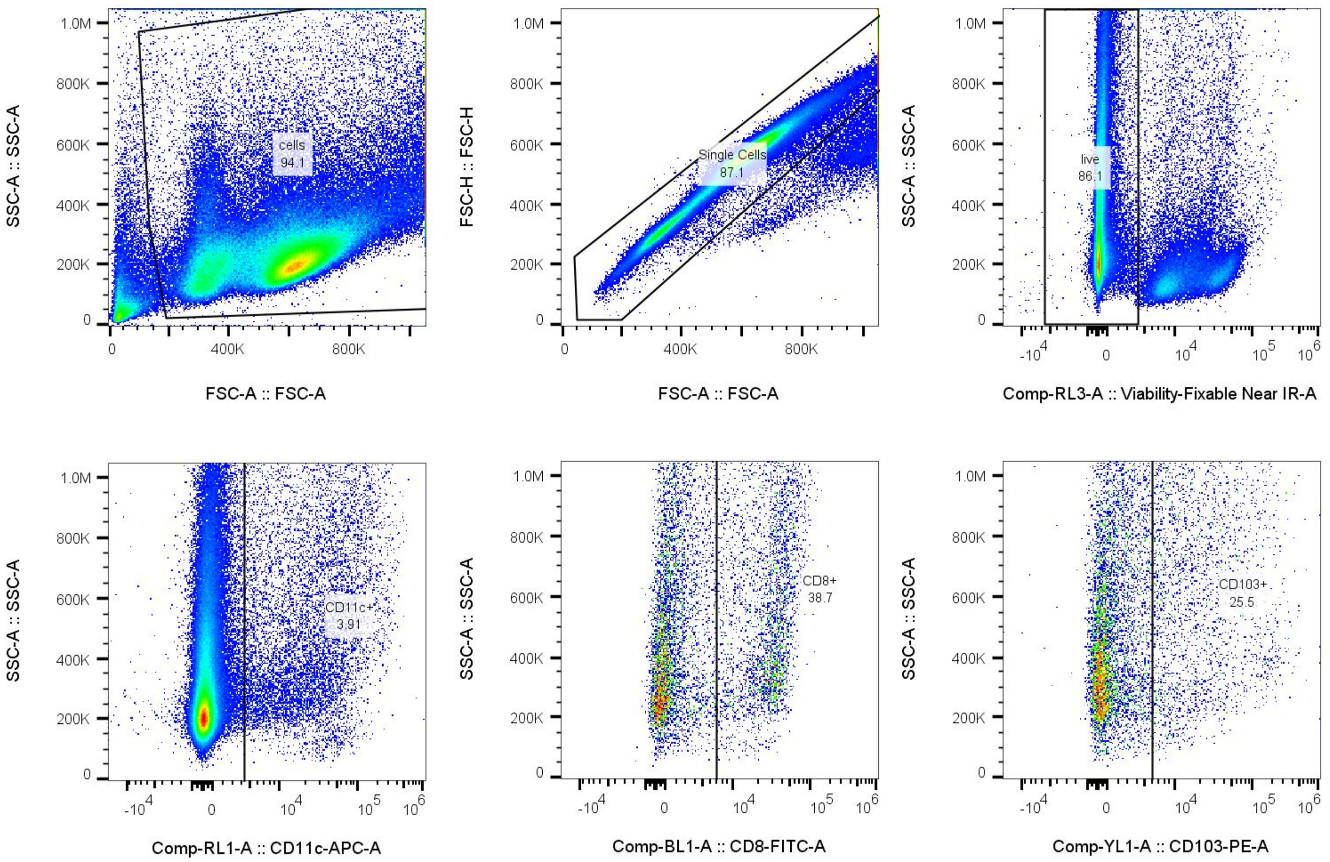

Figure S22. Gating on CD8⁺, CD103⁺ DCs in TDLN.

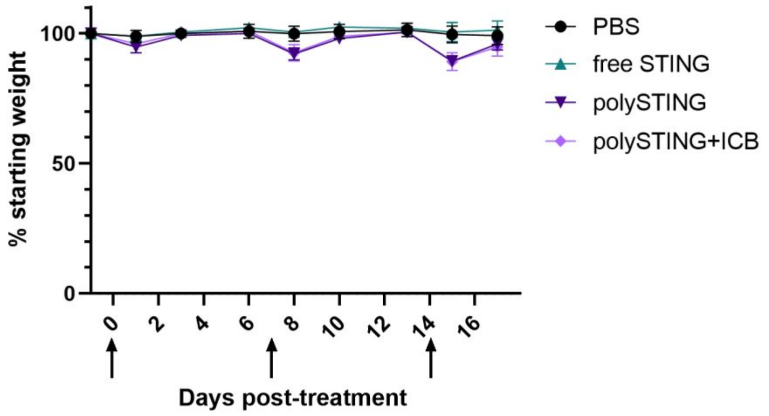

Figure S23. Weight loss in 4T1 study

4. References

(1) Su, F.-Y.; Srinivasan, S.; Lee, B.; Chen, J.; Convertine, A. J.; West, T. E.; Ratner, D. M.; Skerrett, S. J.; Stayton, P. S. Macrophage-Targeted Drugamers with Enzyme-Cleavable Linkers Deliver High Intracellular Drug Dosing and Sustained Drug Pharmacokinetics against Alveolar Pulmonary Infections. *J. Controlled Release* **2018**, *287*, 1–11. https://doi.org/10.1016/j.jconrel.2018.08.014.

(2) Li, S.; Omi, M.; Cartieri, F.; Konkolewicz, D.; Mao, G.; Gao, H.; Averick, S. E.; Mishina, Y.; Matyjaszewski, K. Cationic Hyperbranched Polymers with Biocompatible Shells for siRNA Delivery. *Biomacromolecules* **2018**, *19* (9), 3754–3765. https://doi.org/10.1021/acs.biomac.8b00902.
